# Smart Sorting of Tumor Phenotype with Versatile Fluorescent Ag Nanoclusters by Sensing Specific Reactive Oxygen Species

**DOI:** 10.7150/thno.38422

**Published:** 2020-02-10

**Authors:** Xin Chen, Tiegong Wang, Wenjun Le, Xin Huang, Minghui Gao, Qian Chen, Shuogui Xu, Detao Yin, Qingge Fu, Chengwei Shao, Bingdi Chen, Donglu Shi

**Affiliations:** 1 The Institute for Translational Nanomedicine, Shanghai East Hospital, The Institute for Biomedical Engineering & Nano Science, Tongji University School of Medicine, Shanghai, China.; 2 Radiology Department of Changhai Hospital, The Second Military Medical University, Shanghai, China.; 3 Department of Emergency, Changhai Hospital, Second Military Medical University, Shanghai 200433, China.; 4 The Materials Science and Engineering Program, Dept. of Mechanical and Materials Engineering, College of Engineering and Applied Science, University of Cincinnati, Cincinnati, Ohio, 45221, USA.; 5 Department of Thyroid Surgery, The First Affiliated Hospital of Zhengzhou University, Zhengzhou, Henan 450052, China.

**Keywords:** Ag Nanoclusters, ROS, Tumor Phenotype

## Abstract

Reactive oxygen species (ROS) play a crucial role in cancer formation and development, especially cancer metastasis. However, lack of a precise tool, which could accurately distinguish specific types of ROS, restricts an in-depth study of ROS in cancer development and progression. Herein, we designed smart and versatile fluorescent Ag nanoclusters (AgNCs) for sensitive and selective detection of different species of ROS in cells and tissues.

**Methods**: Firstly, dual-emission fluorescent AgNCs was synthesized by using bovine serum albumin (BSA) to sense different types of ROS (H_2_O_2_, O2•-, •OH). The responsiveness of the AgNCs to different species of ROS was explored by fluorescence spectrum, hydrodynamic diameter, and so on. Furthermore, dual-emission fluorescent AgNCs was used to sense ROS in tumor with different degrees of differentiation. Finally, the relationship between specific types of ROS and tumor cell invasion was explored by cell migration ability and the expression of cell adhesion and EMT markers.

**Results**: This dual-emission fluorescent AgNCs possessed an excellent ability to sensitively and selectively distinguish highly reactive oxygen species (hROS, including O_2_•^-^and •OH) from moderate reactive oxygen species (the form of H_2_O_2_), and exhibited no fluoresence and green fluorescence, respectively. The emission of AgNCs is effective in detecting cellular and tissular ROS. When cultured with AgNCs, malignant tumor cells exhibit non-fluorescence, while the benign tumor emits green and reduced red light and the normal cells appear in weak green and bright red fluorescence. We further verified that not just H_2_O_2_ but specific species of ROS (O_2_•^-^and •OH) were involved in cell invasion and malignant transformation. Our study warrants further research on the role of ROS in physiological and pathophysiological processes.

**Conclusion**: Taken together, AgNCs would be a promising approach for sensing ROS, and offer an intelligent tool to detect different kinds of ROS in tumors.

## Introduction

Cancer metastasis is the leading cause of death worldwide 1-3. Recently, increasing evidence has suggested that excessive generation of reactive oxygen species (ROS) could facilitate tumor formation and progression 4, 5, particularly tumor metastasis 6, 7. A variety of cancers, such as breast, prostate, and thyroid, exhibit excessive and persistent elevation of ROS 8. ROS are comprised of a series of oxidative molecules, such as hydrogen peroxide (H_2_O_2_), superoxide radical (O_2_•^-^), and hydroxyl radical (•OH), which play crucial roles in human physiological and pathophysiological processes 9-11. An increase in ROS production induces severe oxidative stress (OS), leading to damage to biomolecules, such as DNA, proteins, and lipids 11, 12. Cancer cells produce a large amount of ROS, resulting in oxidative damage of DNA to drive tumorigenesis 13-15. Furthermore, elevated levels of ROS generated by cancer cells promote the activity of matrix metalloproteinases (MMP) and epithelial-mesenchymal transition (EMT) 16, 17 and can stimulate tumor migration and invasion. Enhanced ROS levels cause tumor cells to lose adhesion and epithelial polarity, which are critical steps for tumor cell aggressive phenotype transformation 18-21. Three common types of ROS with different oxidant and physical properties participate in not only pathological but also physiological processes. However, it is not clear which type(s) of ROS is involved in tumor cell invasion and malignant transformation. Sensitive and specific detection of various ROS species at intracellular resolution will present significant possibilities for clinical diagnostics of the tumor phenotype. Furthermore, detecting different species of ROS would help explore the underlying mechanisms of tumor initiation and development. There is a critical need to develop a sensitive methodology for accurately distinguishing the specific types of ROS.

Recent advances in nanotechnology have allowed for the detection of ROS measurement at a level of precision previously unachievable 22-30. Wang et al. 22 synthesized water-soluble and highly fluorescent MoS2 quantum dots for detection of hydrogen peroxide (H_2_O_2_) and glucose. Zhang and colleagues 24 developed a single core-shell, nanowire electrodes to measure ROS and RNS in real-time. Yin et al. 25 designed an organic nanoprobe for imaging ROS. Zhou et al. 26 studied a turn-on luminescent probe for monitoring of ROS. Dong and co-workers 27 reported a ratiometric fluorescent probe for imaging of ROS. Enguo's group 28 synthesized heterogeneous nanocomplexes (C-dots-AuNCs) for the detection of ROS. Among these nano-materials, noble metal nanoclusters (NCs) with extremely small sizes, quantum efficiencies, and size-dependent fluorescence are ideal candidates for biolabeling and bioimaging 31, 32. Noble metal nanoclusters (NCs) usually consist of a few to a hundred atoms, whose continuous density of states breaks up into discrete energy levels, exhibiting molecular-like properties 33. Among these, gold nanoclusters (AuNCs) and silver nanoclusters (AgNCs) are the most common types of noble metallic nanoclusters (NCs). Gold nanoclusters (AuNCs) have excellent stable fluorescence properties and low cytotoxicity, and are an ideal candidate for tumor cell and in vivo tumor imaging. Zhang et al.^34^ have developed a red emission(620 nm) Au_20_NCs, which had good targetability for tumor cells and were applied for in vivo tumor imaging.

Silver nanoclusters (AgNCs) are also attractive candidates for bioimaging due to their intrinsic characteristics, diverse fluorescence, and flexible responsiveness 33, 35. The emission of AgNCs can be continuously tuned to cover the entire visible and near-infrared regions, thus accommodating various bioimaging applications. Furthermore, the emission of AgNCs is sensitive to changes in the surrounding environment, making them popular biosensing materials for the detection of ROS. For instance, Liu et al.^36^ used denatured lysozyme protein (dLys) to synthesize dLys- AgNCs with red fluorescence (640 nm), which could be applied to the detection of hydrogen peroxide (H_2_O_2_) and hydroxyl radicals (•OH) in living cells. Li et al.^37^ synthesized oligonucleotide-stabilized AgNCs in situ in a DNA hydrogel, which was demonstrated to be a sensitive and specific probe for cellular imaging and monitoring of ROS/RNS levels. Due to their stable optical properties, AuNCs were always prepared with other fluorescent materials for ROS detection. For example, Chen et al. synthesized nanocomposites with dual fluorescence by combining AuNCs and silicon particles, which could be used for the detection of strong reactive oxygen species^38^; in the presence of hydroxyl radicals (•OH) and hypochlorite (ClO^-^), the orange-red fluorescence of nanocomposites was quenched. Ju et al. 28 combined AuNCs and carbon C-dots as a nano-complex, which have dual fluorescence characteristics and can emit blue fluorescence (450 nm) and orange fluorescence (575 nm). In the presence of hypochlorite (ClO^-^), the orange fluorescence (575 nm) of this complex was diminished while blue fluorescence (450 nm) was unchanged. Although remarkable progress has been made for ROS detection by these previous studies, most of these probes could only detect one kind of ROS, H_2_O_2_, or •OH. There is a lack of a precise probe to accurately distinguishing the specific type of ROS.

**Figure 1:**
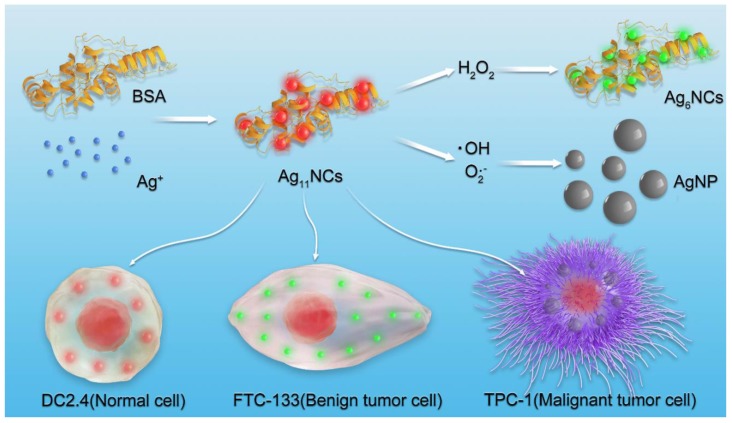
Schematic presentation of the synthesis of AgNCs for selective detection of various species of ROS in cells.

In this study, dual-emission fluorescent AgNCs were designed and developed for sensitive and selective detection of different species of ROS in cells and tissues, as displayed in Figure 1. With the changes in the fluorescence of AgNCs, tumor phenotype could be further judged by the types of ROS. AgNCs are extremely sensitive and selective in distinguishing the highly reactive oxygen species (hROS, including O_2_•^-^and •OH) from moderate reactive oxygen species (the form of H_2_O_2_), which are characterized by either no fluorescence or green fluorescence, while the original AgNCs emit red fluorescence. Since the emission of AgNCs is sensitive and selective to the oxidation state, the fluorescence is effective in detecting cellular and tissular ROS and can be used to distinguish various species of ROS among normal and tumor cells with different degrees of differentiation. More specifically, malignant tumor cells, when cultured with AgNCs, exhibit non-fluorescence, while the benign tumor emits green and reduced red light and the normal cells appear in weak green and bright red fluorescence. The highly reactive oxygen species (hROS, including O_2_•^-^and •OH) are found in malignant tumor cells, but only moderate reactive oxygen species (the form of H_2_O_2_) occur in benign tumor cells. Three commercial fluorescent dyes for detection of diverse species of ROS verified the above results. Besides, three types of antioxidants (neutralization or inhibition of H_2_O_2_, O_2_•^-^and •OH) were employed to verify the validity of AgNCs in the detection of specific ROS. We performed biological studies to confirm the hypothesis that specific species of ROS (O_2_•^-^and •OH) and not H_2_O_2_ drive tumor cell invasion, including cell adhesion and EMT. Taken together, AgNCs exert a versatile role to detect different kinds of ROS in tumors, and provide a logical rationale that the more malignant tumor cells are, the more active species of ROS they contain. AgNCs would, therefore, be a promising approach for sensing ROS, and offer an intelligent tool to explore the role of ROS in physiological and pathophysiological processes.

## Materials and Methods

***Chemicals and reagents:***BSA (Sigma), silver nitrate (AgNO_3_, Sigma), sodium hydroxide (NaOH, Merck), sodium borohydride (NaBH_4_, Merck), and H_2_O_2_ (Merck) were of analytical grade and used without further purification. All other chemicals were of analytical grade and purchased from Sinopharm Chemical Reagent Co., Ltd. (Shanghai, China). All solutions were prepared using ultrapure water, which was obtained through a Millipore Milli-Q water purification system (Billerica, MA, USA), with an electric resistance of 18.3 MΩ. Human thyroid cancer cell lines (FTC-133, B-CPAP, OCUT-2) and murine dendritic cell line (DC2.4) were purchased from the Shanghai Institute of Cell Biology (Shanghai, China). RPMI-1640 Medium (1640), fetal bovine serum (FBS), Phosphate-buffered saline (PBS), and 0.25% Trypsin-EDTA were purchased from Gibco Corp (Grand Island, NY, USA).

***Synthesis of Ag NCs:***For the synthesis of silver nanoclusters (AgNCs), bovine serum albumin (BSA) was used as a stabilizer and sodium borohydride (NaBH_4_) as a reducing agent in an alkaline environment. AgNCs were synthesized following the protocol described by Mathew et al.^39^, with slight modification. First, 5 mL of 10 mM AgNO_3_ solution was added to 5 mL of BSA (0.05 g/mL) solution under vigorous stirring. After 2 min, 0.3 mL of 1 M NaOH was added to this solution under continuous stirring, followed by dropwise addition of 150 μL of 10 mM NaBH_4_. The color of the solution was changed from colorless to reddish-brown, indicating the formation of AgNCs, which were ultrafiltered with a 3 kDa centrifugal ultrafiltration tube to remove unreacted silver ions and NaBH_4_. The ultrafiltered AgNCs solution was centrifuged at 3,500 rpm for 30 min three times. Lastly, the AgNCs solution was passed through a 0.1 μm filter and stored at minus 20°C.

***Physicochemical properties of AgNCs:***The size distribution of AgNCs was explored by using dynamic light scattering (DLS, JEM Zetasizer Nano-ZS90, Great Malvern, England, UK) and high-resolution transmission electron microscopy (HRTEM, JEM-2100F electron microscope, JEOL Ltd., Tokyo, Japan). The fluorescence emission spectra of AgNCs were taken on F-182 4500 spectrophotometer (Hitachi, Chiyoda, Tokyo, Japan). The UV-vis absorption spectra were measured by Cary 50 spectrophotometer (Varian, Palo Alto, CA, USA). Quantitative analysis of silver (Ag) was performed with inductively coupled plasma-atomic emission spectrometry (ICP-AES, P-4010, Hitachi, Chiyoda, Tokyo, Japan). X-ray photoelectron spectroscopy (XPS) experiments were carried out on an RBD upgraded PHI-5000C ESCA system (Perkin Elmer) with Mg Kα radiation (hν=1253.6 eV) to analyze the oxidation state of Ag. The number of the metalcore was measured by matrix-assisted laser desorption ionization time-of-flight mass spectrometer (MALDI TOF MS, ultrafleXtreme MALDI TOF/TOF, Bruker, Germany). Mass spectra were collected in positive mode and averaged for 100 shots. Sinapic acid was used as the matrix for MALDI MS.

***Responsiveness of AgNCs to various species of ROS:***Different types of ROS were detected with the AgNCs. Briefly, 0.5 mL of AgNCs solution was mixed with different types of ROS solution. The concentration of H_2_O_2_ ranged from 0 to 180 μM and hydroxyl radical (•OH) was produced in Fenton reaction (Fe^2+^ + H_2_O_2_ → Fe^3+^ + •OH + OH^-^) by mixing ferrous ions with H_2_O_2_ at a molar ratio of 1:10 at 37 °C for 30 min. The concentration of •OH equaled to that of ferrous ion and ranged from 0 to 50 μM. The superoxide anion (O_2_•^-^) was generated by using the pyrogallol autoxidation method. After incubation of reaction solution for 10 min, the fluorescence spectra, UV-visible (UV-vis) absorption spectra, dynamic light scattering size (DLS), high resolution transmission electron microscope (HRTEM), X-ray photoelectron spectroscopy (XPS) analysis and MALDI-TOF-MS were measured and recorded.

***Stability and photostability of AgNCs:***The stability and photostability of AgNCs at different times were explored. AgNCs were placed under and away from lights for specific times (0 h, 1 h, 2 h, 4 h, 8 h, 12 h) following which the fluorescence and UV-vis absorption spectra of AgNCs were measured and recorded. Also, the stability of AgNCs in other environments (such as metal ions, pH, amino acids cysteine, glutathione) was detected. Briefly, 0.5 mL ultra-filtered AgNCs solution was introduced into 1.5 mL solution with different experimental conditions (such as metal ions, pH, amino acids cysteine, GSH). The concentration of metal ions (Zn^2+^, Fe^3+^, Cu^2+^, Ca^2+^), acids cysteine (Cys), and glutathione (GSH) were 10 μM and pH values ranged from 5 to 9. After incubation of the reaction solution for 30 min, the fluorescence spectra were measured and recorded. Moreover, the stability of AgNCs in GSH with high concentration was explored. Briefly, 0.5 mL ultra-filtered AgNCs solution was added into 1.5 mL GSH solution with different concentrations (2 mM and 5 mM). After incubation of the reaction solution for 15-60 min, the fluorescence spectra were measured and recorded.

***Cell culture:***Human thyroid cancer cell lines (FTC-133, OCUT-2, TPC-1, B-CPAP) and murine dendritic cell line (DC2.4) were maintained at 37 ℃, 5% CO2 in 1640 Medium supplemented with 10% (vol/vol) FBS and 100 UI/mL penicillin and 1 mg/mL streptomycin.

***Biological stability and cytotoxicity assay:***To obtain the biological stability of AgNCs, 0.5 mL ultra-filtered AgNCs solution was introduced into 1.5 ml PBS or RPMI-1640, for specific times (0 h, 1 h, 2h, 4 h, 8 h, 12 h). Subsequently, the fluorescence spectra were measured and recorded. A standard Cell Counting Kit-8 (CCK-8) was utilized to analyze the cytotoxicity of AgNCs following a general protocol. Briefly, murine dendritic cell line DC2.4 was cultured with RPMI-1640 in a 96-well plate with 8×10^3^ cells/well. After incubation at 37°C with 5% CO_2_ and 95% air atmosphere overnight, the AgNCs with concentrations in the range of 0-15mg /mL was added to each well and incubated for specific periods of time (2 h, 4 h, 12 h, 24 h). Cells without AgNCs served as the control group, and the viability was set as 100 %. Next, 10 μL of CCK-8 (5 mg/mL) solution was added to each well of 96-well plate and incubated for another 4 h. The absorbance of each well at 450 nm was measured by using a microplate reader (Tecan infinite M200 Pro, Tecan Group Ltd., Männedorf, Switzerland), and cell viability was determined by the ratio of absorbance of the experimental well to that of the control well. All experiments were performed in triplicate, and the results were averaged.

***Live cell images of cellular ROS with AgNCs:***Human thyroid cancer cell lines (FTC-133, B-CPAP, OCUT-2) and murine dendritic cell line (DC2.4) were grown on 14 mm glass coverslips and allowed to adhere for 12 h. After incubation with 10 mg/mL AgNCs for 1 h, the cells were washed with PBS to remove excess nanoclusters. AgNCs emission images were collected in the range of 450-550 nm (green) and 590-750 nm (red) under excitation of 405 nm. Confocal fluorescence imaging studies were performed with confocal laser scanning microscopy (CLSM, Leica TCS SP5II).

***Tissular ROS imaging with AgNCs:***The animals were anesthetized and the thyroid tumor tissue (OCUT-2), thyroid gland, liver, spleen, and kidney were harvested and cryostat sections were obtained using a microtome. The sections were incubated with 0.2 mL of 10 mg/mL AgNCs for half of an hour. Subsequently, they were washed with PBS to remove excess nanoclusters. AgNCs emission images were collected in the range of 450-550 nm (green) and 590-750 nm (red) under excitation of 405 nm. Confocal fluorescence imaging studies were performed with confocal laser scanning microscopy (CLSM, Leica TCS SP5II).

***FlowSight cellular ROS with AgNCs:*** Human thyroid cancer cell lines (FTC-133, B-CPAP, OCUT-2) and the murine dendritic cell line (DC2.4) were cultured in a 24-well plate at a density of 1×10^5^ cells/well overnight. Subsequently, the cells were incubated with 10 mg/mL AgNCs for 1 h and washed with PBS to remove excess nanoclusters. The cells were digested with trypsin and resuspended in 0.2 mL of PBS. The cell suspension was examined by FlowSight (Merck Millipore, Germany).

***Measurement of cellular ROS by commercial reagents:***Human thyroid cancer cell lines (FTC-133, B-CPAP, OCUT-2) and murine dendritic cell line (DC2.4) were grown on 14 mm glass coverslips and allowed to adhere for 12 h. Cells were then stained with DCHF-DA (10 μM), DHE (100 μM), and APF (20 μM) for 30 min to detect H_2_O_2_, O_2_•^-^, and •OH, respectively. Subsequently, the cells were washed with PBS to remove excess dyes. DCHF-DA and APF emission images were obtained using a 525 nm long-pass filter under excitation using 488 nm, while the emission image of DHE was acquired at 610 nm under excitation using 514 nm. Confocal fluorescence imaging studies were performed with confocal laser scanning microscopy (CLSM, Leica TCS SP5II).

***ROS-blocking imaging with AgNCs:***Human thyroid cancer cell lines (FTC-133, B-CPAP, OCUT-2, TPC-1), and murine dendritic cell line (DC2.4) are grown on 14 mm glass coverslips and were allowed to adhere for 12 h. Cells were pre-cultured in RPMI-1640 in different kinds of ROS-blocking reagents for 2 h, respectively. The ROS-blocking reagents were 500 U/mL CAT (scavenger of H_2_O_2_), 10 mM NAC (scavenging O_2_•^-^), 10 μM DPI (blocking O_2_•^-^), and 1 mM MLT (eliminating •OH). The reagents were dissolved in RPMI-1640. After co-incubation with 10 mg/mL AgNCs for 1 h, the cells were washed with PBS to remove excess nanoclusters. AgNCs emission images were collected in the range of 450-550 nm (green) and 590-750 nm (red) under excitation using 405 nm. Confocal fluorescence imaging studies were performed with confocal laser scanning microscopy (CLSM, Leica TCS SP5II).

***Cell wound scratch assay:***Briefly, Human thyroid cancer cell lines (FTC-133, B-CPAP, OCUT-2, and TPC-1) and murine dendritic cell line (DC2.4) were cultured in 6-well plates at a density of 1×10^6^cells/well until cells reached 95% confluence. A cell-free area was created by scratching confluent cells with yellow-tip. After incubating for 0, 12, 24, and 36 h, the images of scratched cells were taken under a phase-contrast microscope. The cell-free area was analyzed using the software Image J.

***Cell wound scratch assay after ROS blocking:***Human anaplastic thyroid cancer cell line OCUT-2 was grown in a 6-well plate at a density of 1×10^6^ cells/well until cells reached 95% confluence. The cells were further cultured with RPMI-1640 containing different ROS scavengers, respectively. CAT (500 U/mL), NAC (10 mM), and MLT (1 mM) were added to the culture medium to neutralize H_2_O_2_, O_2_•^-^ and •OH, respectively. Subsequently, a cell-area was created by scratching the confluent cells with a yellow tip. After incubating with CAT, NAC, and MLT for 0, 12, 24, and 36 h, the images of scratched cells were taken under a phase-contrast microscope. The cell-free area was analyzed by software Image J.

***RT-PCR assay:***Expression of NOX-4, E-cadherin, and MMP-9 mRNAs in human thyroid cancer cell lines (FTC-133, B-CPAP, OCUT-2, and TPC-1) were analyzed by RT-PCR. Total RNA was extracted with Trizol (Invitrogen). One microgram of total RNA was used for Thermoscript RT-PCR according to the Invitrogen procedure manual. The primer sequences for NOX-4, E-cadherin, and MMP-9 are presented in Table 1.

***Immunofluorescence assay:***Immunofluorescence assay was performed as instructed in the Abcam procedure manual. Human thyroid cancer cell lines (FTC-133, B-CPAP, OCUT-2, TPC-1) were grown on 14 mm glass coverslips and allowed to adhere for 12 h. Then the cells were fixed with pre-chilled 4% paraformaldehyde for 15 min, permeabilized with 0.25% Triton X-100 in PBS for 10 min, and blocked with 10% goat serum for 30 min. After blocking, the cells were treated with rabbit monoclonal anti-NOX4 (1: 1000), rabbit monoclonal anti-E-cadherin (1: 200), or rabbit monoclonal anti-MMP-9 (1: 200) at 4 °C overnight. After washing with PBS, cells were incubated with Cy3 or FITC-labeled goat anti-rabbit antibody (dilution ratios 1:100 and 1:200, respectively) at room temperature for 1 h in the dark. The cell nuclei were counterstained with DAPI for 5 min. The fluorescence images were taken by confocal microscopy (Leica).

## Results and Discussion

The results from this study and previous researches provide persuasive evidence that ROS contributes to the formation and progression of cancer, especially tumor metastasis 40-42. Excessive levels of ROS have been detected in several tumors, such as breast, ovarian, and prostate cancers 43-48. These studies suggested that increased ROS generation is likely to drive oncogenic transformation 49-51. NOX-generated ROS has been proved to facilitate malignant transformation through EMT and MMP activity 52-54. The effects of oxidative stress may rely on the types of ROS involved and their levels and duration. For example, Sudjit et al. found that instead of H_2_O_2_, the up-regulated •OH promoted tumor cell migration and invasion 49. However, the production of ROS is tightly controlled both spatially and temporally within the cell, making it difficult to sensitively and selectively detect ROS. A possible solution for the detection of ROS may be provided by noble metallic nanoclusters (NCs) with intrinsic fluorescence for bioimaging 55. In this study, we designed and developed dual-emission fluorescent AgNCs for sensitive and selective detection of different species of ROS in cells and tissues.

### Synthesis and characterization of AgNCs

**Figure 2:**
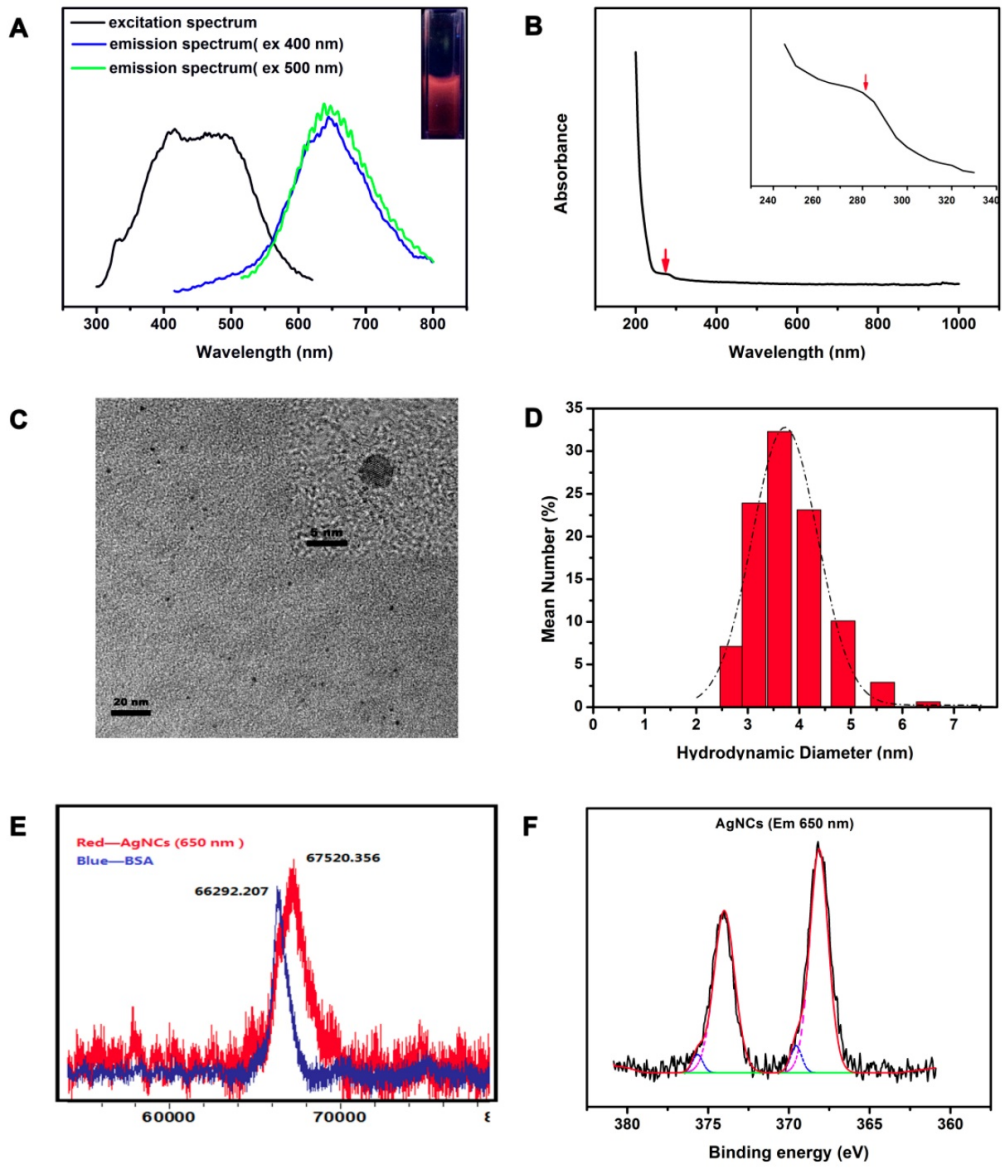
** Characterization of AgNCs. (A)** Fluorescence spectrum of AgNCs. The black line represents the excitation spectrum of AgNCs; blue and green lines represent the emission spectrum under excitation at 400 and 500 nm, respectively. The inset photograph was pictured under UV light. **(B)** UV-Vis spectrum of AgNCs. The inset photograph is enlargement of 240 to 340 nm absorption wavelength. **(C)** TEM and HRTEM images of AgNCs. **(D)** Hydrodynamic diameter of AgNCs detected by DLS. **(E)** Mass peaks of BSA and AgNCs measured by MALDI-TOF-MS. Blue and red lines show the mass peaks of BSA and AgNCs, respectively. **(F)** XPS spectrum of AgNCs. The original and composite spectra are in black and red, respectively. The pink line represents Ag (0) binding energy.

For selective detection of different species of ROS, AgNCs with extraordinary luminescent properties were synthesized according to a simple and rapid method, as illustrated in Figure 1. Typically, the BSA and silver nitrate solutions were mixed vigorously for 2 min and NaOH solution was then added to the mixture. Subsequently, NaBH_4_ solution was added dropwise until the color of the solution changed into claret-red. AgNCs were formed with red fluorescence under UV light (Figure 2). The optical properties of AgNCs were characterized by UV-Vis and fluorescence spectrum.

As shown in Figure 2A, AgNCs exhibited a broad excitation spectrum (ranging from 350 nm to 600 nm) with peaks at 400 nm and 500 nm. The emission spectrum did not change significantly, with an emission peak at 650 nm when excited at 400 nm and 500 nm, except for the origin of emission spectrum. The position of fluorescence emission peak remained unchanged with various excitation wavelengths indicating the intrinsic fluorescence. The UV-Vis absorption spectrum of AgNCs (Figure 2B) had a strong, featureless decayed absorption in the UV region and a decreasing absorption peak at 274 nm. The absence of metal surface plasmon resonance (SRP) peak (400-500 nm) in the AgNCs UV-Vis spectrum excluded the existence of silver nanoparticles (AgNPs) in the AgNCs solution.

The HRTEM image (Figure 2C) shows a uniform and monodisperse sphere of AgNCs with an average diameter of around 4 nm. The inset shows the lattice feature of AgNCs. Figure 2D displays the DLS results showing the hydrodynamic diameter of AgNCs ranging from 2.5 nm to 5 nm, with a peak of 3.615 nm. This size range is comparable to the Fermi wavelength of conduction electrons, resulting in discrete electronic transitions and size-dependent fluorescence. The metal nuclearity of AgNCs were quantified by MALDI-TOF-MS (Figure 2E). The mass peak of BSA was located at 66.29 kDa, while that of AgNCs shifted to 67.52 kDa. This difference corresponded to 11 atoms of Ag, as the metal core in AgNCs. Notably, the 4 nm size of AgNCs detected by HRTEM appeared to be bigger than that of Ag_11_NCs. The TEM investigations have not been successful for nobel metal nanoclusters, because clusters in the size range of 1 nm undergo electron-beam-induced coalescence, leading to bigger particles 33. The tendency of cluster collapse upon irradiation was prominent for Ag clusters. As reported previously, the TEM diameters of Ag_15_NCs@BSA^39^ and Ag_14_NCs@HSA^56^ were around 5 and 4 nm, respectively.

The XPS measurements showed two groups of Ag 3d peaks of AgNC de-convoluted into two components, as shown in Figure 2F. In the main component, a group of Ag 3d peaks was located at 368.18 eV (Ag 3d5/2) and another at 374.33 eV (Ag 3d3/2). The energy gap between the two peaks was nearly 6.0 eV, confirming the presence of Ag (0) in AgNCs. In another component, two other peaks were located at 369.03 eV (Ag 3d5/2) and 375.18 eV (Ag 3d3/2), indicating the presence of Ag (I) in AgNCs.

Since the formation of AgNCs is related to the amount of reducing agent, by controlling the addition of NaBH_4_, the fluorescence properties of intermediate stages during the synthesis were investigated. As shown in Figure S1, the fluorescence intensity of excitation spectrum increased with the concentration of NaBH_4_, with the peak position unchanged; same was true for the emission spectrum with excitation wavelength at 500 nm. However, when excited at 400 nm, the fluorescence intensity and peak position changed with the increasing concentration of NaBH_4_. Consistently, the fluorescence intensity intensified with the peak position shifted from 500 nm to 650 nm.

### Responsiveness of AgNCs to different species of ROS

**Figure 3:**
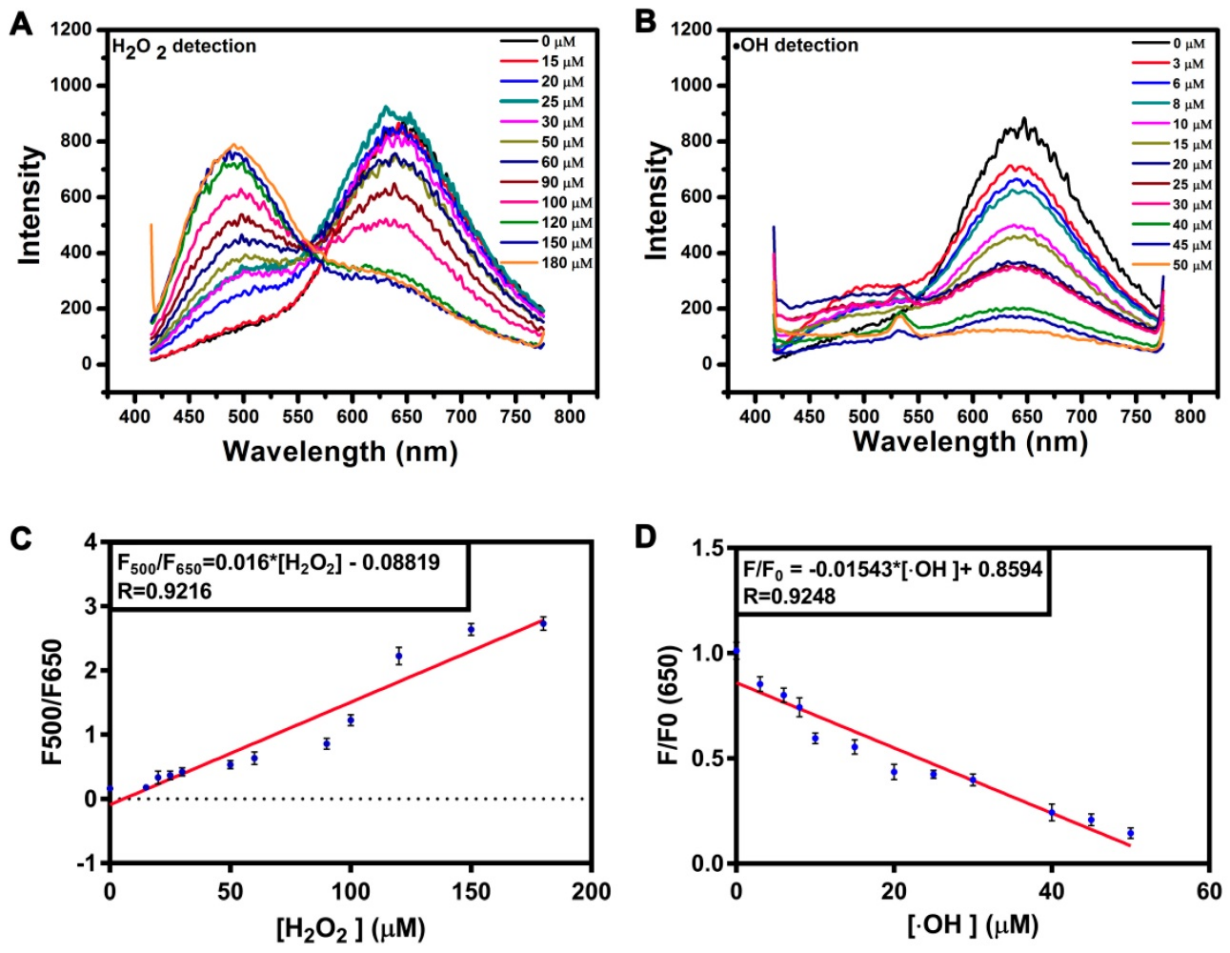
** Responsiveness of AgNCs to various species of ROS. (A)** FL emission changes of AgNCs excited at 400 nm with an increased amount of H_2_O_2_. **(B)** FL emission changes of AgNCs excited at 400 nm with an increased amount of •OH. **(C)** Dose-response of H_2_O_2_ on AgNCs FL emission. The F500 and F650 are fluorescence emission intensities at 500 and 650 nm. **(D)** Dose-response of •OH on AgNCs FL emission. The F and F0 are fluorescence emission intensities in the absence and presence of •OH, respectively.

The responsiveness of the AgNCs to different species of ROS is shown in Figure 3. With the addition of different types of ROS (H_2_O_2_, O_2_•^-^, •OH), the fluorescence intensity and emission peak band changed dissimilarly (excited at 400 nm). H_2_O_2_, a moderate ROS, could tune AgNCs emission from red (650 nm) to green (500 nm). With increasing H_2_O_2_, the red emission (650 nm) of AgNCs was quenched gradually, while the green emission (500 nm) was enhanced (Figure 3 A). The fluorescence intensity ratio of 500 nm to 650 nm (F500/F650) was linearly proportional to the concentration of H_2_O_2_ (Figure 3C). However, upon adding highly active ROS (O_2_•^-^, •OH), the red emission (650 nm) weakened dramatically, but the green emission (500 nm) did not emerge (Figure 3B and Figure S2). The red emission (650 nm) decreased linearly with increasing hydroxyl radicals (•OH) (Figure 3D).

**Figure 4:**
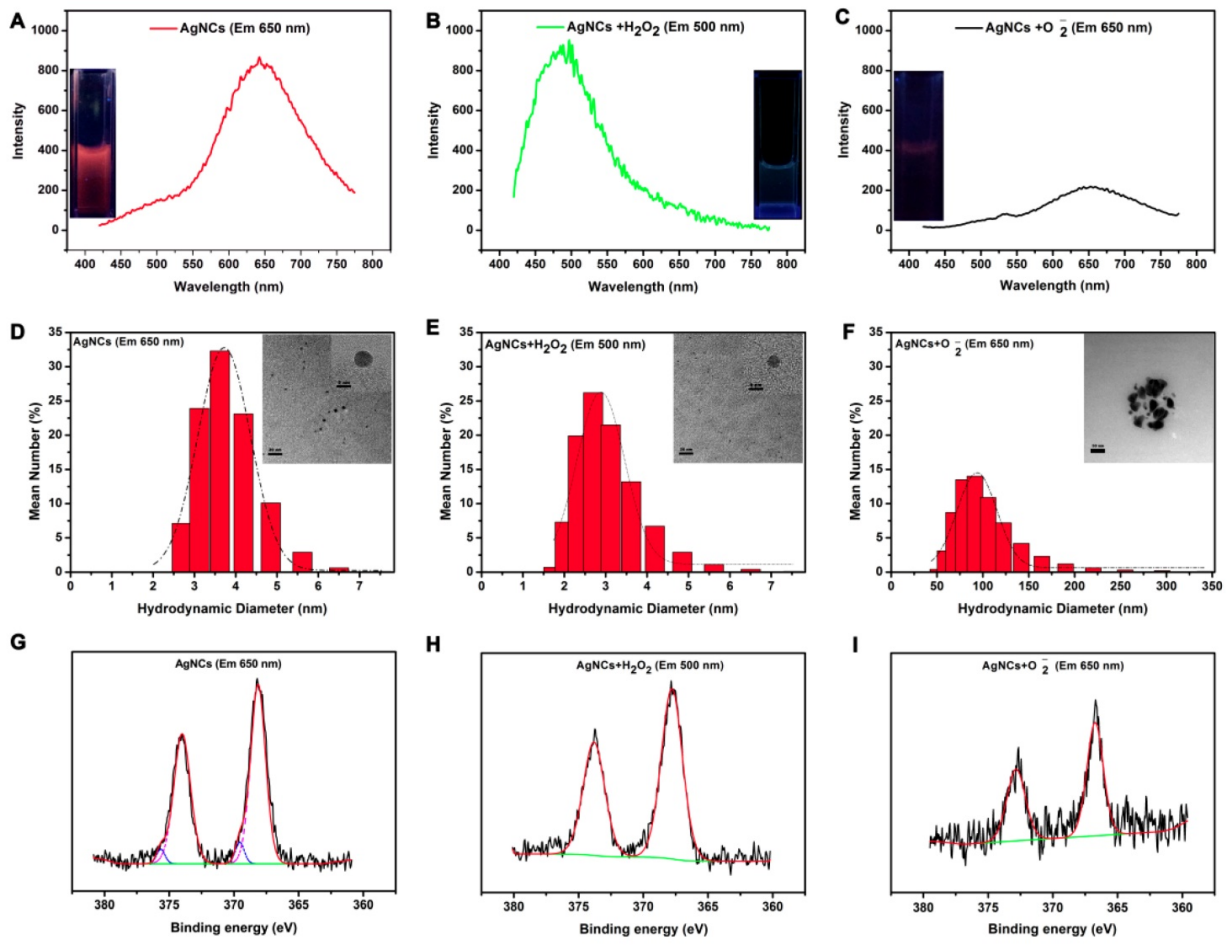
** Mechanism of AgNC responsiveness to various species of ROS.** Emission spectra and photographs under UV light (inset) for AgNCs **(A)**, AgNCs with H_2_O_2_** (B)**, and AgNCs with O_2_•^-^ **(C)**. Hydrodynamic diameter and HRTEM image (inset) for AgNCs **(D)**, AgNCs with H_2_O_2_** (E)**, and AgNCs with O_2_•^-^ **(F)**. XPS spectrum of AgNCs **(G)**, AgNCs with H_2_O_2_** (H)**, and AgNCs with O_2_•^-^ **(I)**.

As shown in Figure 4, the solutions of original AgNCs, AgNCs with H_2_O_2_, and AgNCs with O_2_•^-^ exhibited red, green, and no fluorescence, respectively, under UV light. Figure 4A-B show the emission peaks of AgNCs and AgNCs with H_2_O_2_ solutions located at 650 nm and 500 nm, respectively. The AgNCs with O_2_•^-^solution had a weak emission peak at 650 nm (Figure 4C). As shown in Figure S3A, AgNCs had a decreasing absorption peak at 274 nm, which is the characteristic absorption of BSA. AgNCs with H_2_O_2_ solution only exhibited a strong, featureless decayed absorption in the UV region. However, AgNCs with O_2_•^-^solution had three absorption peaks at 274 nm, 350 nm, and 450 nm. The absorption peak at 450 nm represented the metal SRP peak, indicating the formation of silver nanoparticles in AgNCs with O_2_•^-^ solution. The UV-Vis results provided evidence for the ROS-mediated AgNCs fluorescence that was linked to their size.

As shown in the HRTEM image (Figure 4D), the diameter of AgNCs was around 4 nm. The size of AgNCs with H_2_O_2_ was also homogeneous but smaller than that of AgNCs, with a reduced diameter of 3 nm (Figure 4E). The silver nanoparticles were found in AgNCs with O_2_•^-^, whose diameter had reached 50 nm (Figure 4F). Consistently, the DLS results showed the hydrodynamic diameter of AgNCs between 2 nm and 5 nm, with the largest proportion of 3.615 nm (Figure 4D). The sizes of AgNCs with H_2_O_2_ ranged between 2 nm to 4 nm, with a peak of 2.696 nm (Figure 4 E). The fluorescence of AgNCs with O_2_•^-^ was significantly reduced due to larger hydration particle size between 50 nm and 150 nm, with the largest proportion of 91.28 nm (Figure 4F).

These results indicated that AgNCs behaved like molecular species due to size-dependent fluorescence. As shown in Figure S3B, MALDI-TOF-MS analysis on the mass peaks of AgNCs (red emission) and AgNCs with H_2_O_2_ (green emission) at 67.52 kDa and 66.98 kDa, respectively. This indicated that the red-emitting of AgNCs and green-emitting of AgNCs with H_2_O_2_ corresponded to Ag_11_NCs and Ag_6_NCs with masses of 1.23kDa and 0.69 kDa. The differences in the oxidation state of the metal-core were also detected among AgNCs, AgNCs with H_2_O_2_ and AgNCs with O_2_•- based on the XPS results (in Figure 4 G-I). For AgNCs, the Ag3d peaks (including 3d5/2 and Ag 3d3/2) were decomposed into two parts: the first: 368.13 eV (Ag 3 d5/2) and 374.0 eV (Ag 3 d3/2), whose energy gap between the two peaks was 6.0 eV consistent with Ag (0); the second: 369.15eV (Ag 3d5/2) and 375.65 eV (Ag 3d3/2), whose binding energy was characteristic of Ag (1). For AgNCs with H_2_O_2_, the 3d5/2 and Ag 3d3/2 peaks were located at 367.74 eV and 373.75 eV, respectively. The energy level difference between the two peaks was 6.0 eV, confirming the presence of Ag (0) in AgNCs with H_2_O_2_. The XPS result of AgNCs with O_2_•^-^ was similar to that of AgNCs with H_2_O_2_, whose Ag 3d binding energies were 366.70 eV and 372.65 eV, respectively. The binding energy gap was 6.0 eV, corresponding to Ag (0). The variation in the oxidation state was attributable to the fluorescence profiles. The moderate ROS (H_2_O_2_) could lead to a reduction of the metal core, while AgNC agglomeration was associated with the highly active ROS.

### Stability and photo-stability of AgNCs

**Figure 5:**
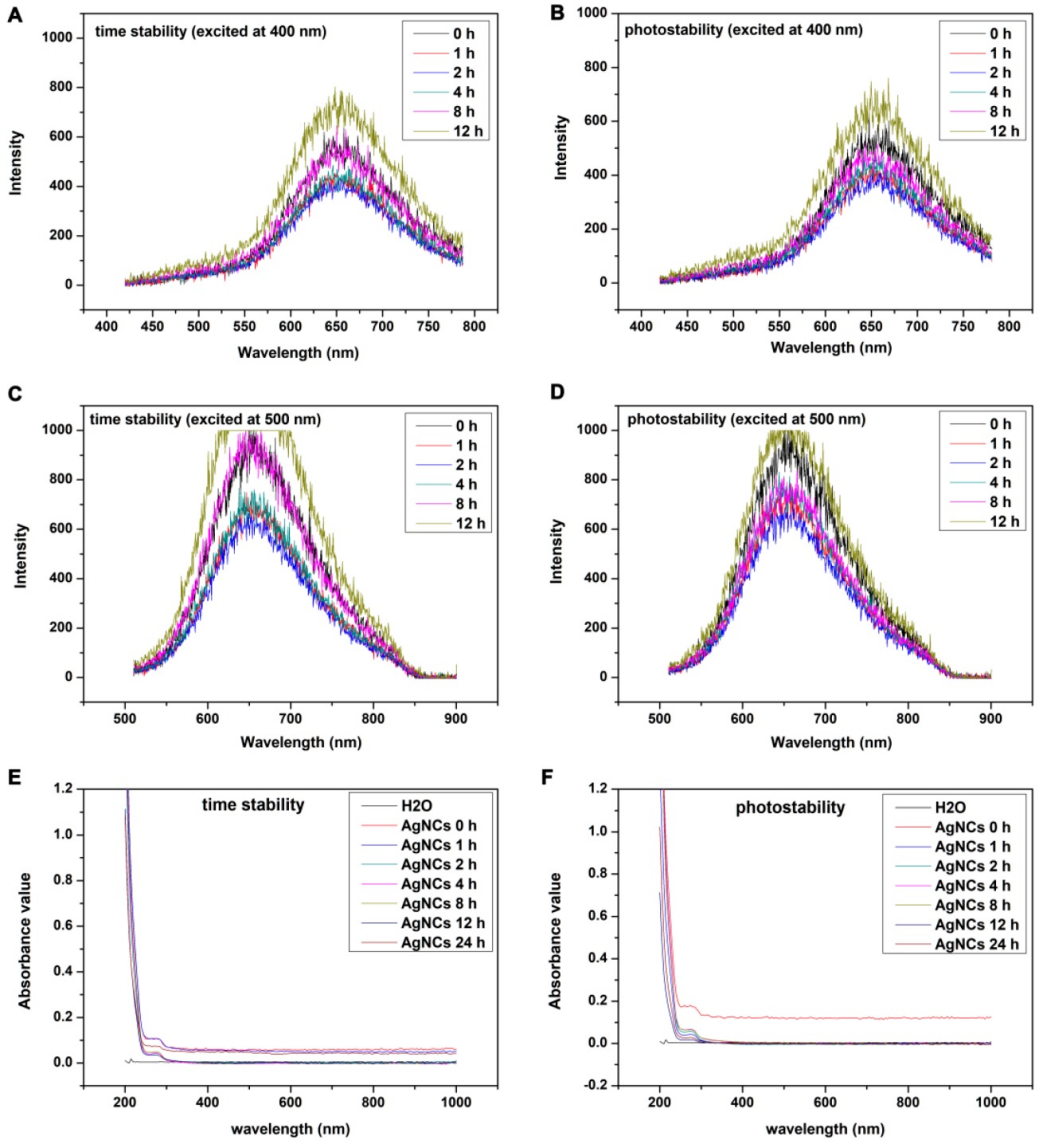
** Time-stability and photo-stability of AgNCs.** Emission spectrum of AgNCs excited at 400 nm under lights** (A)** and away from lights **(B)**. Emission spectrum of AgNCs excited at 500 nm under lights **(C)** and away from lights **(D)**. UV-vis absorption spectra of AgNCs under lights **(E)** and away from lights **(F)**.

Fluorescence and the UV-vis absorption spectrum were used to explore the time-stability and photo-stability of AgNCs. As shown in Figure 5, the results of time-stability and photo-stability of AgNCs were similar. The emission spectrum of AgNCs excited at 400 nm under lights (Figure 5A) but away from lights the fluorescence intensity of AgNCs slightly declined within 8 h (Figure 5B). The fluorescence intensity at 12 h began to go up but there was no shift of fluorescence emission peak. The changes of emission spectrum excited at 500 nm under lights (Figure 5C) and away from lights (Figure 5D) were similar to that excited at 400 nm. Furthermore, UV-vis absorption spectra of AgNCs under lights (E) and away from lights (F) were unchanged, indicating that there was no obvious formation of silver nanoparticles (AgNPs). Also, the stability of AgNCs in other environments (such as metal ions, pH, cysteine, GSH) were detected, as shown in Figure S4. It is evident from Figure S4A, the fluorescence intensity of AgNCs excited at 400 nm was relatively stable with Zn^2+^ and Cu^2+^ at a concentration of 10 μM, but declined with Fe^3+^ and Ca^2+^ at a concentration of 10 μM. Furthermore, the fluorescence intensity of AgNCs excited at 400 nm was relatively stable with 10 μM Cys and GSH. Also, the emission spectrum of AgNCs excited at 400 nm was stable at pH values from 5 to 7 but declined at pH 8 and 9; however, there was no shift of fluorescence emission peak.

As previously reported 26, 57, GSH is elevated in tumor microenvironment with a concentration in the range of 2.0 to 10.0 mM, whereas its value in normal cells is only about 1/4 of that in tumor cells. We have explored the stability of AgNCs in GSH with high concentration. As shown in Figure S5, the fluorescence intensity of AgNCs excited at 400 nm was enhanced in the presence of GSH and there was a green fluorescence emission peak of AgNCs in 5 mM GSH for 1 h (Figure S5B). These results indicated that AgNCs were relatively stable at a low concentration of GSH. When incubated with GSH with a concentration of 5 mM, the fluorescence emission peak of AgNCs changed. However, there was no green fluorescence of AgNCs in tumor cells which might be due to the oxidative stress of malignant tumors. Although the concentration of GSH in tumor cells is about 4 times of that in normal cells, ROS in tumor cells is also much higher than in normal cells^4^. Furthermore, a method to synthesize stable AgNCs in situ in DNA Hydrogel has been described 37, which will help us to improve the stability of AgNCs.

### Biological stability and cytotoxicity of AgNCs

**Figure 6:**
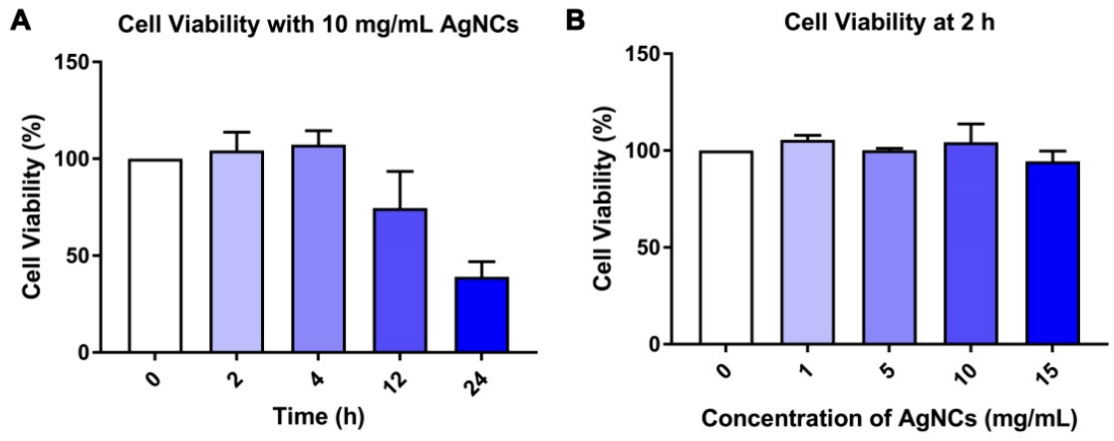
** Biological toxicity of AgNCs. (A)** Cell viability of DC2.4 cells after treatment with 10 mg/mL AgNCs for specific times (2 h, 4 h, 12 h, 24 h).** (B)** Cell viability of DC2.4 cells incubated with 0-15 mg/mL AgNCs for 2 h.

For the biomedical application of AgNCs, it is necessary to evaluate their stability in bio-fluids and in vitro cytotoxicity. As shown in Figure S6, the fluorescence intensity of AgNCs excited at 400 nm and 500 nm slightly declined in PBS (Figure S6A-B) and RPMI-1640 (Figure S6C-D). CCK-8 assay was used to investigate the cell viability of murine dendritic cells (DC2.4) incubated with 0-15 mg/mL AgNCs for 24 h. Figure 6A shows that when cultured with 10 mg/mL AgNCs for 12 h, the viability of DC 2.4 cells was inhibited. Negligible cell death was observed when DC2.4 cells were incubated with 0-15 mg/mL AgNCs for 2 h (Figure 6B), which was sufficient time to measure cellular ROS using AgNCs without cytotoxicity.

### Living cell images of cellular ROS with AgNCs

**Figure 7:**
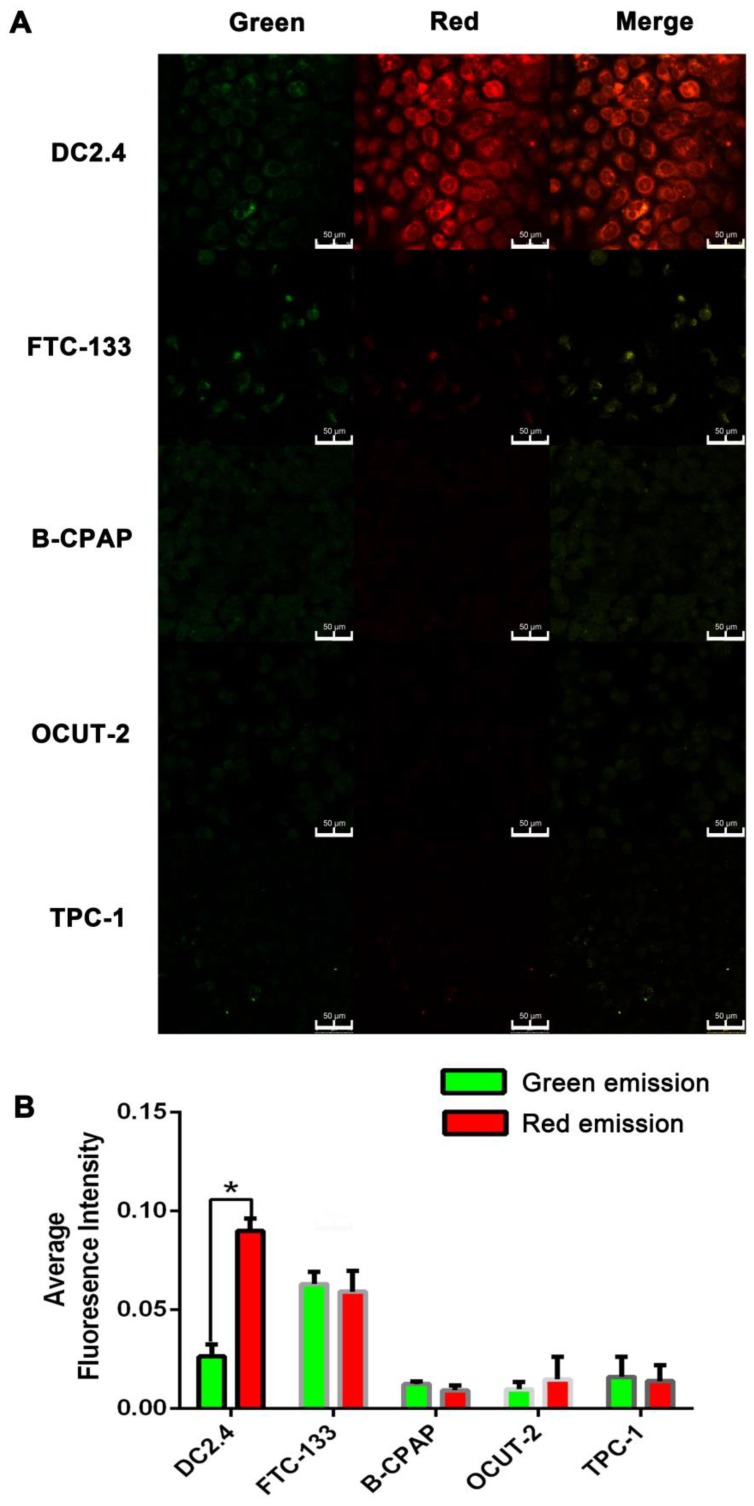
** Live cell images of cellular ROS with AgNCs. (A)** Confocal images of cellular ROS with AgNCs in dendritic cells (DC2.4) and thyroid cancer cell lines (FTC-133, B-CPAP, OCUT-2, TPC-1). **(B)** Average red fluorescence intensity of dendritic cells (DC2.4) and thyroid cancer cell lines (FTC-133, B-CPAP, OCUT-2, TPC-1).

The tumor cells with different stages of differentiation and normal cells were chosen for validation of AgNCs in the detection of cellular ROS. Briefly, cells were co-cultured with 10 mg/mL AgNCs for 1 h, and washed with PBS twice for confocal imaging. Thyroid cancer cell lines (FTC-133, B-CPAP, OCUT-2, TPC-1) and dendritic cells (DC2.4) were initially used to detect cellular ROS with AgNCs. There was a marked difference in fluorescence changes between thyroid tumor cells and dendritic cells. As displayed in Figure 7A, DC2.4 cell exhibited slight green and bright red fluorescence, merging to become an orange fluorescence. However, in thyroid tumor cells, red fluorescence was significantly reduced, suggesting the presence ROS in tumor cells. In benign follicular thyroid tumor cells (FTC-133 cell), green fluorescence intensified with decreased red fluorescence compared to DC 2.4 cells and the merged image exhibited yellow fluorescence. The responsiveness of AgNCs to different ROS levels was evident by enhanced green fluorescence attributable to H_2_O_2_ in FTC-133with a moderate ROS level. As for malignant thyroid tumor cells (B-CPAP, OCUT-2, TPC-1), both green and red fluorescence were dramatically quenched, indicating that these malignant thyroid tumor cells contain relatively active ROS (such as O_2_•^-^ and •OH). Accordingly, the average red fluorescence intensity in DC 2.4 cell was the highest, and the average green fluorescence in FTC-133 was increased (Figure 7B). Furthermore, for malignant thyroid tumor cells (B-CPAP, OCUT-2, and TPC-1), both green and red fluorescence were dramatically weakened.

AgNCs were also used to detect cellular ROS in other types of tumor cells with various grades of differentiation, including ovarian, breast, and prostate cancers. Similar results were obtained from confocal images (Figure S 7, 8, and 9). Differentiated tumor cells (A2780, MCF-7, Lincap), like FTC-133, exhibited enhanced green fluorescence and reduced red fluorescence. However, in malignant tumor cells (including skov3, ES2, MDA-MB-231, DU-145, PC-3), both green and red fluorescence were significantly quenched, which was consistent with the results of malignant thyroid tumor cells. These results lead to a rational hypothesis that diverse species of ROS exist in normal cells and tumor cells with different degrees of differentiation.

### FlowSight cellular ROS with AgNCs

**Figure 8:**
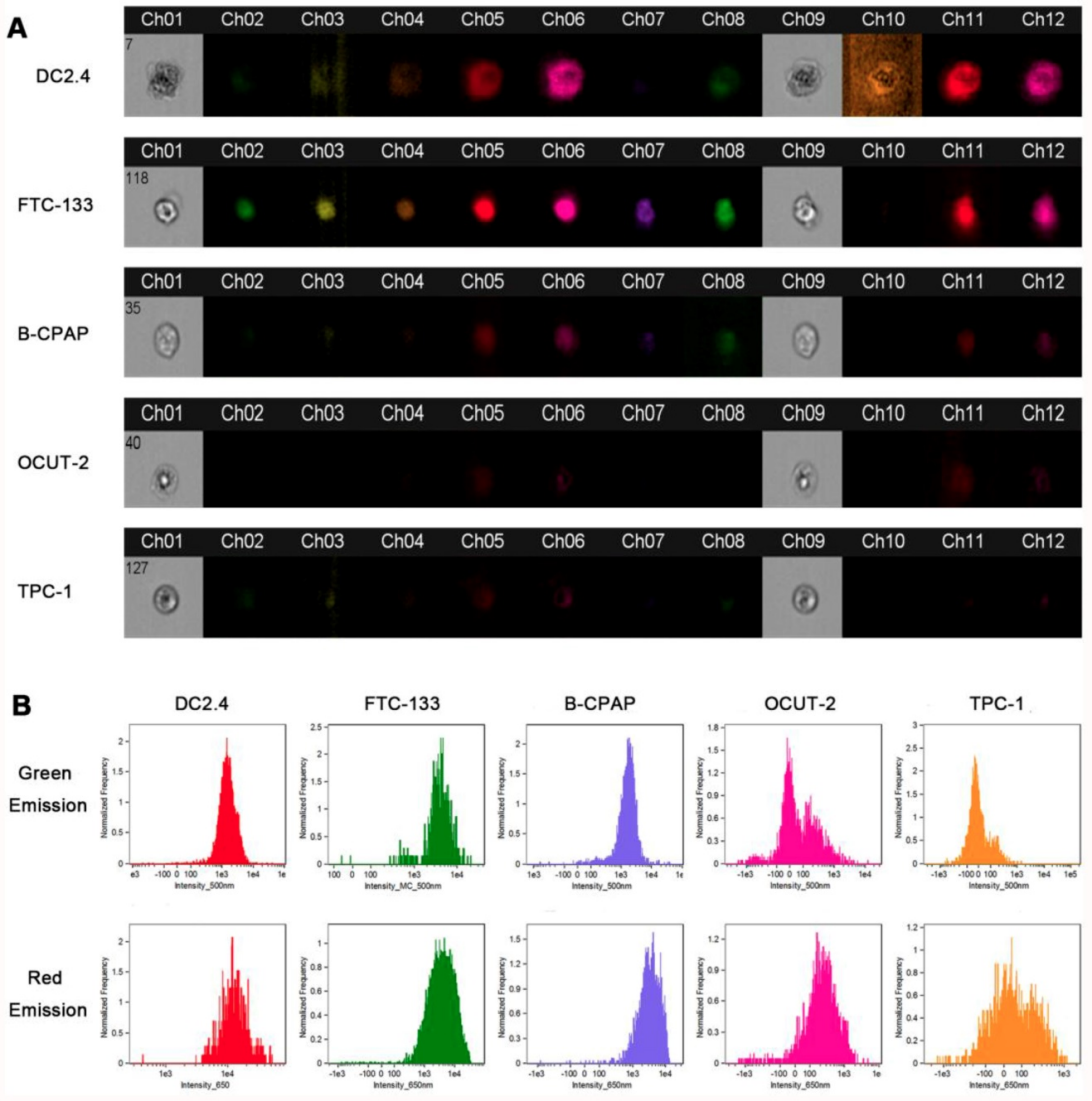
** FlowSight cellular ROS with AgNCs. (A)** FlowSight image of cellular ROS with AgNCs in dendritic cells (DC2.4) and thyroid cancer cell lines (FTC-133, B-CPAP, OCUT-2, TPC-1). The ch07 and ch08 can check blue (435-505 nm) and green (505-560 nm) fluorescent signal under ultraviolet light (405 nm). The cho9 is in charge of bright field. **(B)** ROS levels in dendritic cells (DC2.4) and thyroid cancer cell lines (FTC-133, B-CPAP, OCUT-2, TPC-1) measured by FlowSight cytometry with AgNCs.

FlowSight was carried out by using AgNCs in thyroid cancer cell lines (FTC-133, B-CPAP, OCUT-2, TPC-1) and dendritic cells (DC2.4), enabling imaging, count, and classification of cells based on optical and fluorescence techniques. Both **ch07** and **ch08** were used to validate blue (435-505 nm) and green (505-560 nm) fluorescent signals under ultraviolet light (405 nm), while **cho9** was employed for bright field. The orange (595-642 nm) and red (642-745 nm) fluorescence images were observed under excitation at 405 nm. Figure 8A displays optical and fluorescent images of dendritic cells (DC2.4) and thyroid cancer cell lines (FTC-133, B-CPAP, OCUT-2, TPC-1). Dendritic cells (DC2.4) emitted bright red and slight green signals and an adjacent orange fluorescence could be detected. In the benign follicular thyroid tumor cell line FTC-133, however, there was no orange signal while the red fluorescence weakened. Simultaneously, the green emission signal was quite strong and the adjacent blue fluorescence was absent. Both red and green emissions were apparently reduced in B-CPAP, a poorly differentiated thyroid carcinomal cell line. Further, in anaplastic thyroid tumor cell lines (OCUT-2 and TPC-1), the red and green fluorescence signals were almost faded. The red fluorescence intensity in DC 2.4 was the highest, and the green fluorescence in FTC-133 significantly increased, as shown in Figure 8B. Furthermore, for malignant thyroid tumor cells (B-CPAP, OCUT-2, TPC-1), not only green but also red fluorescence intensity was distinctly reduced. This experimental evidence showed the presence of various species of ROS in normal cells and thyroid cancer cells with different degrees of differentiation.

### ROS-blocking imaging with AgNCs

**Figure 9:**
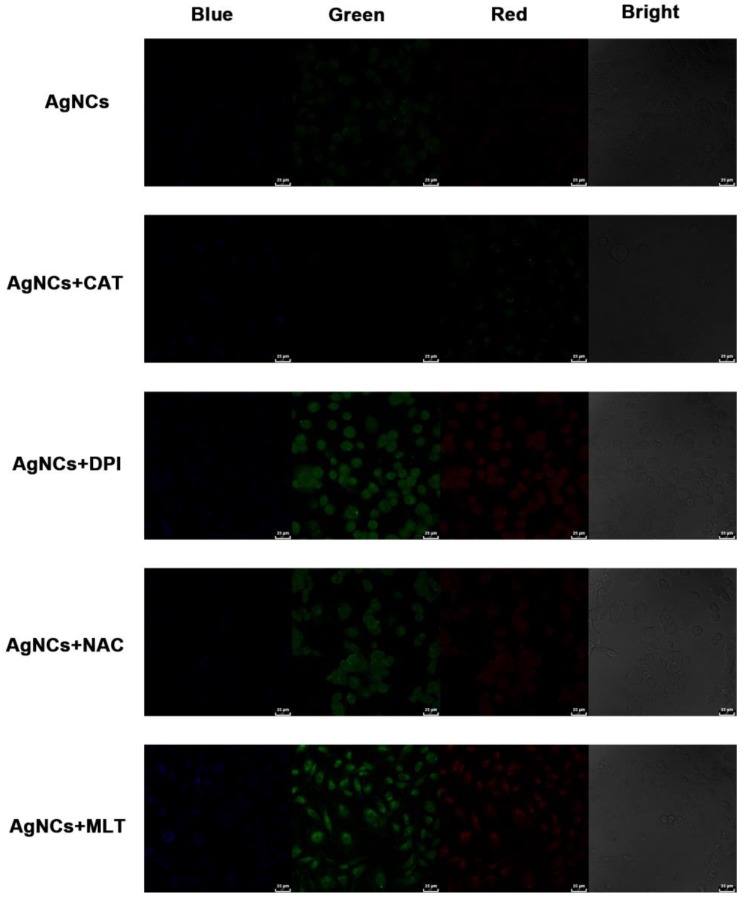
** ROS-blocking imaging with AgNCs.** Malignant thyroid carcinoma cells (OCUT-2) cultured in RPMI-1640 with various ROS-blocking agents (CAT, DPI, NAC and MLT). Cellular ROS was detected by confocal imaging with AgNCs.

To further investigate various ROS species in differentiated tumor cells, the experiments were performed of sensing cellular ROS after blocking and detection of cellular ROS with commercial reagents. A malignant thyroid carcinoma cell line, OCUT-2, was cultured in the RPMI-1640 with the ROS-blocking agents for reverse verification. H_2_O_2_ could be decomposed by catalase (CAT), while diphenyliodonium chloride (DPI, a Nox inhibitor) and N-acetyl-L-cysteine (NAC, an antioxidant) were used for blocking the generation of O_2_•^-^ while melatonin (MLT, an antioxidant) could eliminate •OH^58^, ^59^. No apparent changes were observed after CAT blocking (Figure 9), suggesting that H_2_O_2_ did not cause a decrease in green and red fluorescence. Upon blocking O_2_•^-^ by DPI or NAC, the green fluorescence signal was enhanced along with red fluorescence. Furthermore, when OCUT-2 cells were cultured with MLT to scavenge •OH, both green and red emission signals were increased, showing active species of ROS (O_2_•^-^ and •OH) well distributed in the malignant thyroid cancer cells resulting in decreased green and red fluorescence.

### Measurement of cellular ROS by commercial reagents

**Figure 10:**
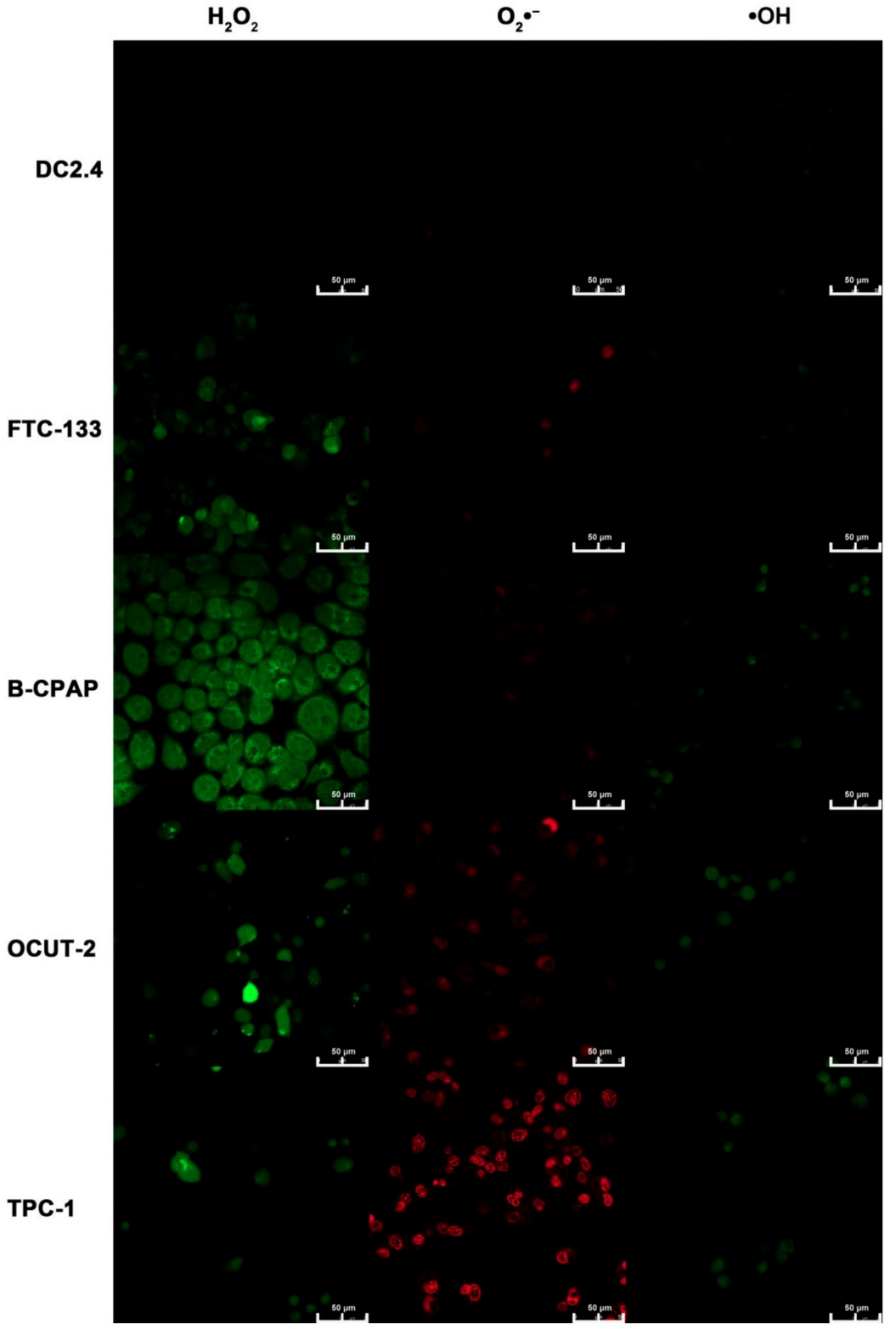
** Measurement of cellular ROS by commercial reagents.** Cellular ROS of dendritic cells (DC2.4) and thyroid cancer cell lines (FTC-133, B-CPAP, OCUT-2, TPC-1) are detected by commercial reagents DCHF-DA, DHE, and APF for the detection of H_2_O_2_, O_2_•^-^, •OH, respectively.

In addition to the reverse verification experiment, a positive verification experiment was performed by using commercial reagents. DCHF-DA, DHE, and APF are primarily used for the detection of H_2_O_2_, O_2_•^-^, •OH, respectively. Almost no fluorescence for H_2_O_2_, O_2_•^-^, and •OH was observed in DC 2.4 cells, as shown in Figure 10. As for FTC-133 cells, both green fluorescence for H_2_O_2_ and weak red fluorescence for O_2_•^-^ were observed; the green fluorescence was identified for H_2_O_2_, while faint red fluorescence indicated the presence of O_2_•^-^. B-CPAP cells exhibited two different fluorescence signals for H_2_O_2_ and •OH. For malignant thyroid carcinoma cell lines (OCUT-2 and TPC-1), the green of •OH and red of O_2_•^-^ became intensified, especially the latter in the TPC-1 cells. The results from the verification experiment supported the hypothesis that diverse species of ROS were present in both normal and tumor cells, but with different degrees of differentiation. Thus, the presence of more active species of ROS would imply the existence of more malignant tumor cells.

### Tissular ROS imaging with AgNCs in various tissues

**Figure 11:**
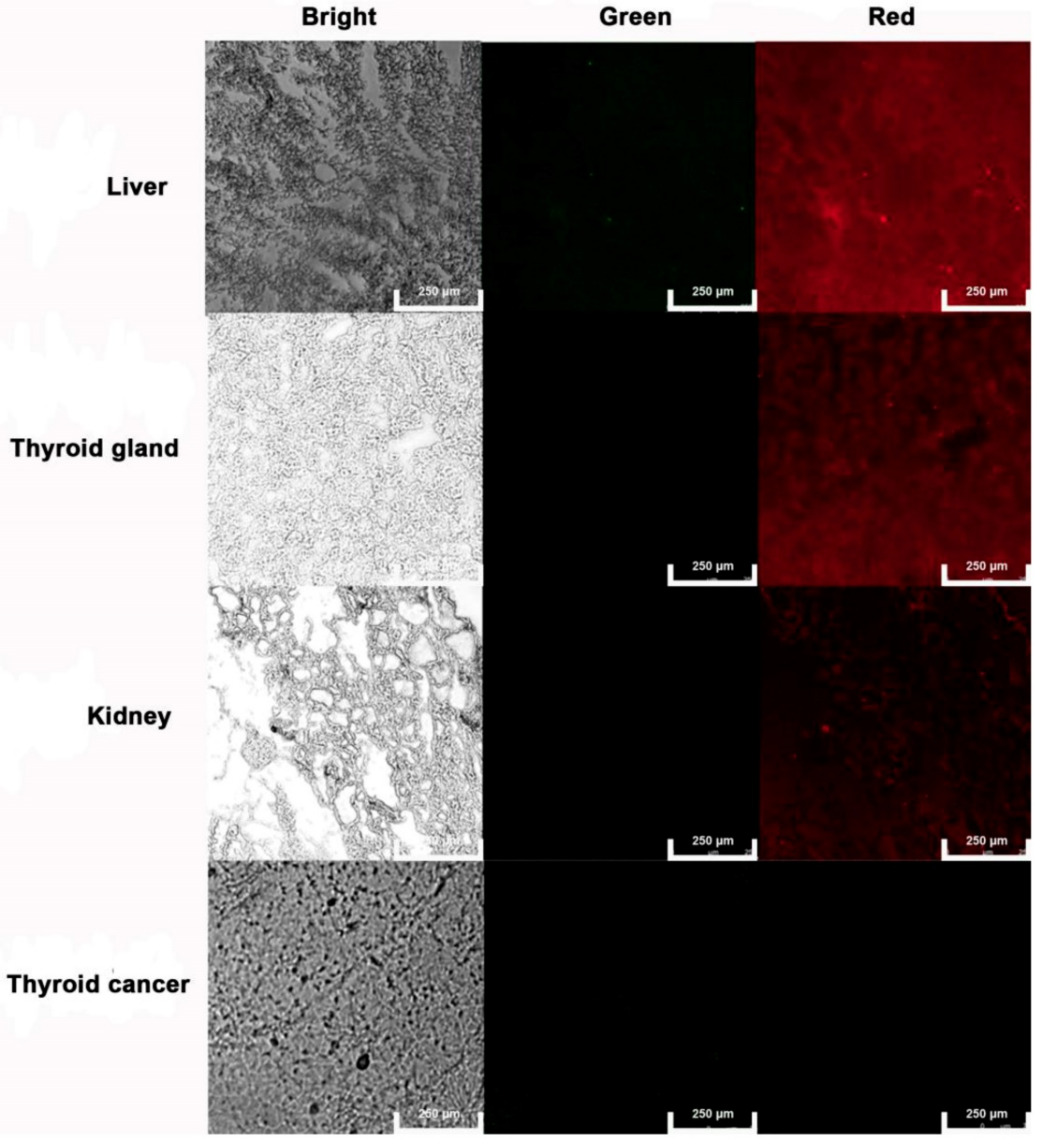
** Tissular ROS imaging with AgNCs.** Frozen sections of the liver, kidney, thyroid gland, and anaplastic thyroid cancer tissues were used to sense tissular ROS with AgNCs.

The liver, kidney, thyroid gland, and anaplastic thyroid cancer tissues were sectioned and frozen for AgNCs sensing of tissular ROS. As shown in Figure 11, the liver exhibited weak green and bright red fluorescence, indicating production of H_2_O_2_. Both kidney and thyroid gland display relatively weak red fluorescence. As a hormone synthesis organ, the thyroid gland activates more ROS to generate thyroxine. Moreover, neither green nor red fluorescence signal was observed in anaplastic thyroid tissue, confirming the presence of different species of ROS in both normal and tumor tissues.

### The mechanism of ROS in malignant tumor phenotype

**Figure 12:**
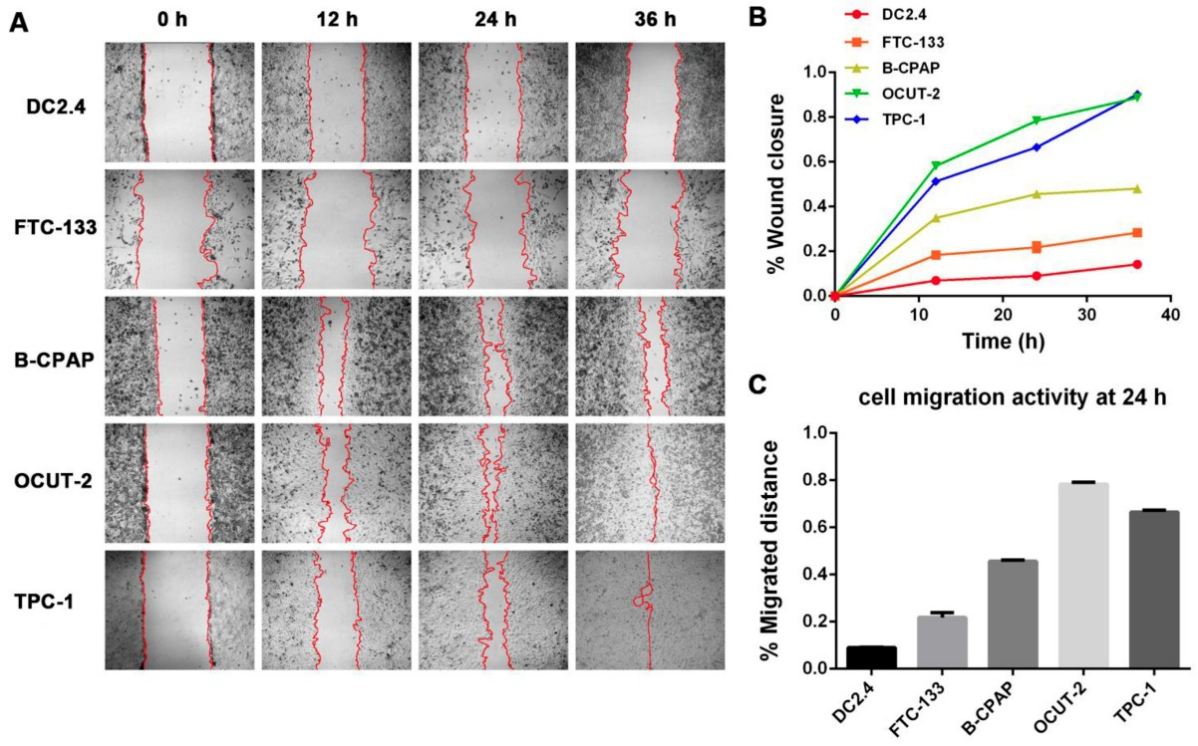
** Migration ability of dendritic cells and thyroid cancer cells with various grades of differentiation. (A)** Cell wound scratch assay of dendritic cells (DC2.4) and thyroid cancer cells with various grades of differentiation (FTC-133, B-CPAP, OCUT-2, TPC-1). **(B)** Migration speed of dendritic cells (DC2.4) and thyroid cancer cells with various grades of differentiation (FTC-133, B-CPAP, OCUT-2, TPC-1). **(C)** percent migrated distance of the five cell lines (DC 2.4, FTC-133, B-CPAP, OCUT-2, TPC-1) at 24 h.

Based on the above experimental results, we concluded that the aggressiveness of tumor cells was proportional to the presence of active species of ROS. The migration ability of dendritic cells (DC2.4) and thyroid cancer cells with various grades of differentiation (FTC-133, B-CPAP, OCUT-2, TPC-1) were investigated by cell wound scratch assay (Figure 12). Figure 12A-B show that anaplastic thyroid cancer cells (OCUT-2 and TPC-1) had high migration capacity for tumor metastasis. The migration ability of poorly differentiated thyroid cancer cell line (B-CPAP) was relatively moderate. The migration of differentiated follicular thyroid tumor cells (FTC-133) was found to be slow compared to that of the malignant thyroid cancer cell lines. The migration ability of dendritic cell (DC2.4) was the lowest among the five cell lines. Figure 12C displays the percentage of the migrated distance of the five cell lines (DC 2.4, FTC-133, B-CPAP, OCUT-2, TPC-1) at 24 h. The migrated distance of OCUT-2 was the longest, while that of DC 2.4 was the shortest. Table S1 summarizes the histologic characteristics and genetic alterations of tumor cells. The migrated distance was directly associated with the aggressiveness of tumor cells. Based on these results, we conclude that active species of ROS is the typical characteristic of aggressive tumor cells and a hallmark of the malignant tumor phenotype.

**Figure 13:**
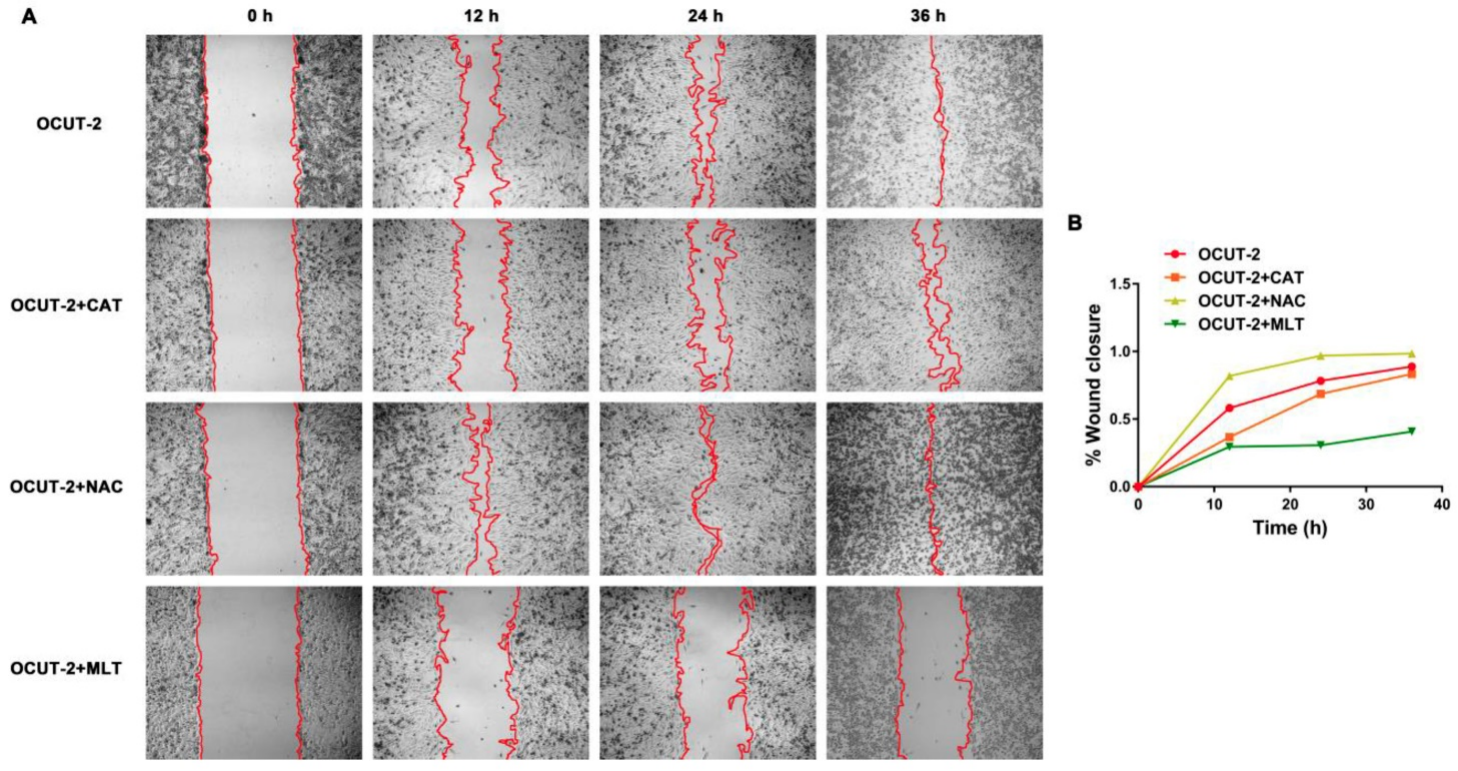
** Effect of ROS on the migration ability of OCUT-2 anaplastic thyroid tumor cells. (A)** Cell wound scratch assay of OCUT-2 in RPMI-1640 with various ROS-blocking agents (CAT, NAC, and MLT). **(B)** Migration of OCUT-2 in the presence of various ROS-blocking agents (CAT, NAC, and MLT).

The effect of ROS on migration ability of anaplastic thyroid tumor cells (OCUT-2) was investigated. Figure 13A shows the migration activity of OCUT-2 in the RPMI-1640 medium with ROS-blocking reagents (CAT, NAC, MLT). The migration activity of OCUT-2 varied with specific ROS blocking reagents: it was not affected by NAC (blocking O_2_•^-^) and was effectively inhibited by MLT (scavenging •OH). After adding CAT and MLT, the migration speed of OCUT-2 declined. The significant slow-down in the migration speed of OCUT-2 cells was attributable to the highly active ROS (•OH) triggering migration in the malignant tumor cells.

**Figure 14:**
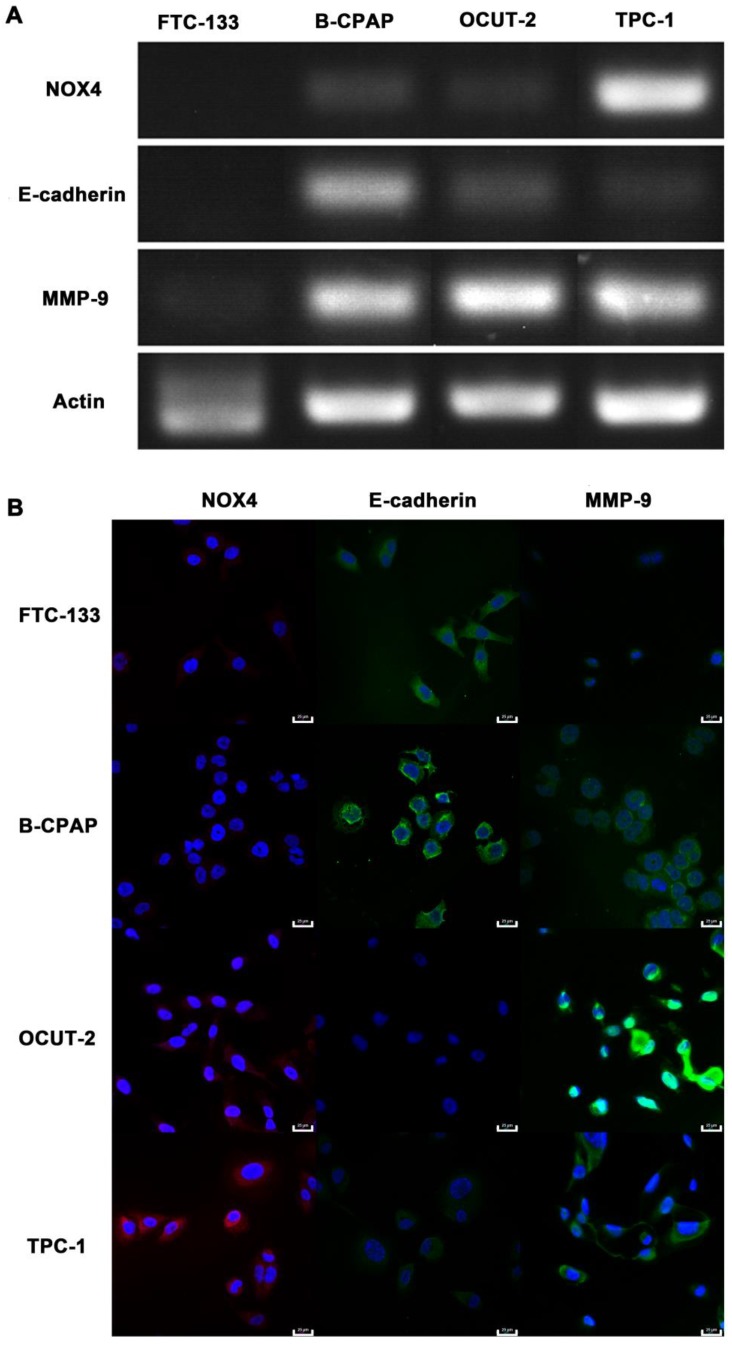
** Genes related to malignant phenotype (E-cadherin and MMP-9) and ROS production (NOX4) in thyroid cancer cells. (A)** mRNA levels of E-cadherin, MMP-9, and NOX4 in thyroid cancer cells with various grades of differentiation (FTC-133, B-CPAP, OCUT-2, TPC-1) detected by RT-PCR. **(B)** Protein levels of E-cadherin, MMP-9, and NOX4 in thyroid cancer cells with various grades of differentiation (FTC-133, B-CPAP, OCUT-2, TPC-1) measured by immunofluorescence.

The genes related to the malignant phenotype (E-cadherin and MMP-9) and ROS production (NOX4) were also studied in thyroid cancer cells with various grades of differentiation (FTC-133, B-CPAP, OCUT-2, TPC-1). Down-regulated E-cadherin is a hallmark of EMT, a complex process during which epithelial cells loose intercellular adhesion, with fibroblast-like characteristics and migratory and invasive properties 60, 61. The up-regulated metalloproteinases MMP-9, can degrade the extracellular matrix and lose cell adhesion for tumor progression 35. NOX4 is the most frequently expressed NOX isoform for its relevance to cancer and is a major source of ROS production 62-64. The mRNA expression of NOX4, E-cadherin, and MMP-9 were detected by PCR (Figure 14A). Neither E-cadherin nor MMP-9 was expressed in FTC-133 while both were expressed in B-CPAP. As for anaplastic thyroid tumor cells (OCUT-2 and TPC-1), E-cadherin expression was significantly decreased and MMP-9 expression was distinctly increased. Figure 14B shows the immunofluorescence staining of thyroid cancer cells, revealing the protein level of NOX4, E-cadherin, and MMP-9. At the protein level, FTC-133 expressed E-cadherin, while B-CPAP expressed both E-cadherin and MMP-9. For anaplastic thyroid tumor cells (OCUT-2 and TPC-1), E-cadherin was not expressed, while the expression of NOX4 and MMP-9 was enhanced, especially in OCUT-2. Thus, down-regulated E-cadherin and up-regulated NOX4 and MMP-9 were detected in anaplastic thyroid cancer cells (OCUT-2 and TPC-1). These results indicated that NOX4-produced ROS was responsible for the migratory and invasive properties of tumor cells by regulating the expression of E-cadherin and MMP-9.

## Conclusions

In this study, we developed dual-emission fluorescent Ag nanoclusters (AgNCs) to sensitively and selectively detect various species of ROS in cells and tissues. AgNCs with ultra-small size, under excitation of 400 or 500 nm, emitted red fluorescence peaking at 650 nm. When exposed to various species of ROS, there was a sensitive and varied response of fluorescence of AgNCs. Moderate ROS (H_2_O_2_) shifted the emission peak of AgNCs switching from red (650 nm) to green (500 nm). The highly active ROS (O_2_•^-^, •OH) resulted in a dramatic descent of red emission (650 nm). The reaction mechanism of AgNCs in response to ROS was identified by the size management and oxidation state of the metal core as AgNCs exhibited size-dependent fluorescence changes. AgNCs with green emission (500 nm) revealed smaller size after exposure to H_2_O_2_, while the addition of highly active ROS (O_2_•^-^, •OH) led to the aggregation of AgNCs subsequently quenching fluorescence. The MALDI-TOF-MS results confirmed green- and red- emitting AgNCs to be Ag_6_NCs and Ag_11_NCs, respectively. The green- and non- emitting AgNC metal core behaved like Ag(0), while the oxidation state of red-emitting AgNCs contained not only Ag(0) but also Ag(1).

AgNCs possess versatile functions for live images of cellular and tissular ROS that could be observed by confocal microscopy and Flowsight. These live image results confirmed AgNCs' ability to selectively and sensitively distinguish specific species of ROS. When AgNCs were applied to tumor cells of different pathologic grades and normal cells, tumor cells with varying degrees of differentiation were found to contain various species of ROS. The normal cell line (DC2.4) exhibited slight green, bright red fluorescence, and merged orange fluorescence. The red fluorescence signal in tumor cells declined significantly, while the intensity of green fluorescence relied on the grades of differentiation of tumor cells. These results provided evidence that AgNCs are a good candidate for cellular and tissular ROS detection. However, it is worth noting that AgNCs, as silver nanomaterials, also have a drawback for ROS detection as they could induce toxicity by modulating ROS in cells. As reported previously, GSH-Ag^0^NCs induce higher cellular toxicity via the modulation of ROS when compared to GSH-Ag^+^NCs^65^. Therefore, when applying AgNCs to detect cellular and tissular ROS, the concentration of and incubation time with AgNCs during ROS detection must be strictly controlled to avoid cytotoxicity. In this study, 10 mg/mL AgNCs was a safe concentration for cellular and tissular ROS detection without cytotoxicity.

Differentiated thyroid tumor cells (FTC-133) exhibited enhanced green emission, implying the presence of H_2_O_2_. No fluorescence was observed in anaplastic thyroid tumor cells, indicating the production of highly active ROS (O_2_•^-^, •OH) in malignant thyroid cancer cells. Therefore, we concluded that more malignant tumor cells produce more active species of ROS. With ROS blocking reagents (CAT, DPI, NAC, MLT), the fluorescence signal of AgNCs could be detected in the malignant thyroid carcinoma cells (OCUT-2). When cultured with MLT (scavenging •OH), both green and red emission signals were enhanced. The results of cellular ROS detected by commercial reagents were consistent with those measured by AgNCs. The migration assay also revealed that highly active ROS (•OH) might facilitate migration of malignant tumor cells (OCUT-2). Thus, the type of ROS species might be correlated with the aggressive phenotype of tumor.

## Supplementary Material

Supplementary figures and table.

## Figures and Tables

**Table 1:** Summary of primers sequence.

Gene	Primer sequence (5'-3')
H-ACTIN-S	CACCCAGCACAATGAAGATCAAGAT
H-ACTIN-A	CCAGTTTTTAAATCCTGAGTCAAGC
H-NOX4-S	ATTTAGATACCCACCCTCCCG
H-NOX4-A	CACAGTACAGGCACAAAGGTCC
H-MMP9-F	CACATCAATTTAGGGACAAAGAGC
H-MMP9-R	CTTCCGCAGGCTGAATCTTC
H-E-CAD(RZ)-S	CAACAAAGACAAAGAAGGCAAGG
H-E-CAD(RZ)-A	TGAGAGAAGAGAGTGTATGTGGC
