# Supplementary material for: Smart Sorting of Tumor Phenotype with Versatile Fluorescent Ag Nanoclusters by Sensing Specific Reactive Oxygen Species
Source: Theranostics. 2020 Feb 10;10(8):3430–50. doi: 10.7150/thno.38422 (PMC7069096; doi:10.7150/thno.38422)
Supplement: Supplementary file 1 — Supplementary figures and table. [file thnov10p3430s1.pdf]

Supplementary Materials

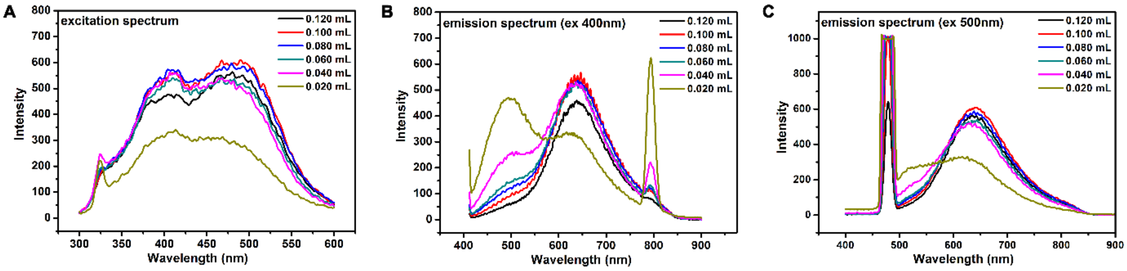

Figure S1. Fluorescent intensity changes with increased concentration of $NaBH_4$. (A) Excitation spectrum of AgNCs with increasing concentration of $NaBH_4$. **(B)** Emission specturm of AgNCs excited at 400 nm with increasing concentration of $NaBH_4$. **(C)** Emission specturm of AgNCs excited at 500 nm with increasing concentration of $NaBH_4$.

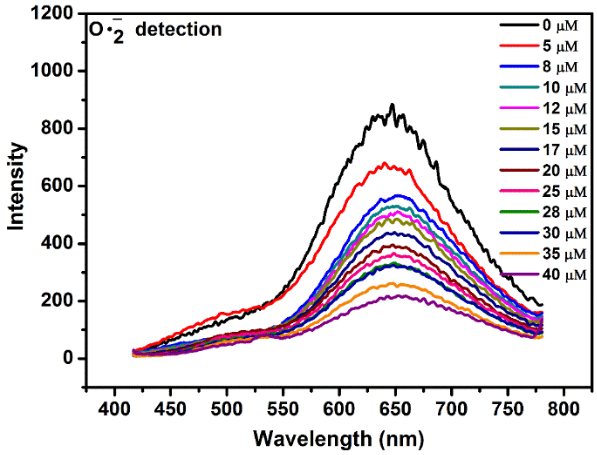

Figure. S2 FL emission changes of AgNCs (excited at 400 nm) with $O_2\bullet^-$solution.

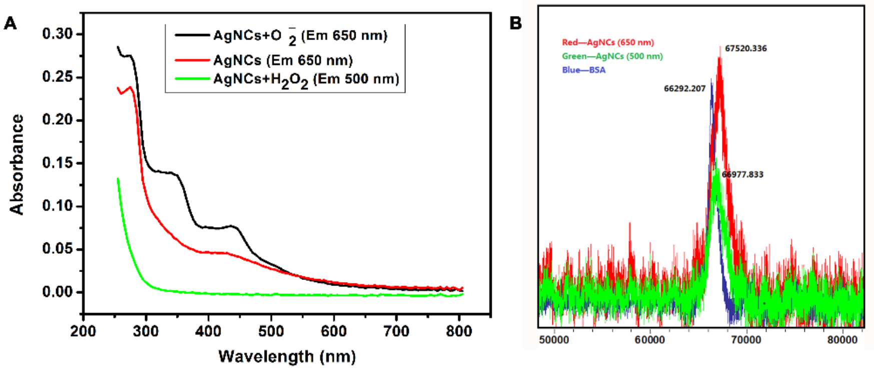

Figure S3. UV-Vis spectra and Mass Peaks of AgNCs (A) UV-Vis spectra of AgNCs with O_2^-, AgNCs, AgNCs with H_2O_2. **(B)** Mass peaks of BSA, AgNCs, and

AgNCs with H_2O_2. Blue, Green, and Red lines exhibit the mass peaks of BSA, AgNCs with H_2O_2, and AgNCs, repectively.

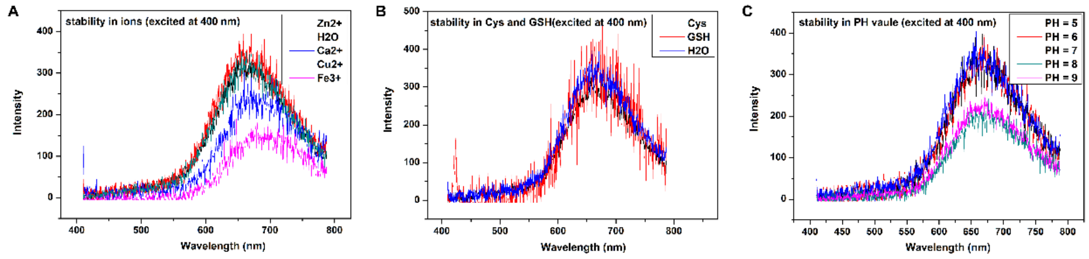

Figure S4. **Stability of AgNCs in various environments (such as metal ions, pH, amino acids cysteine, GSH).** Emission spectrum of AgNCs excited at 400 nm in the presence of metal ions **(A)** and amino acids **(B)**. Emission spectrum of AgNCs excited at 400 nm at different pH **(C)**. The concentration of metal ions and amino acids were 10 μM.

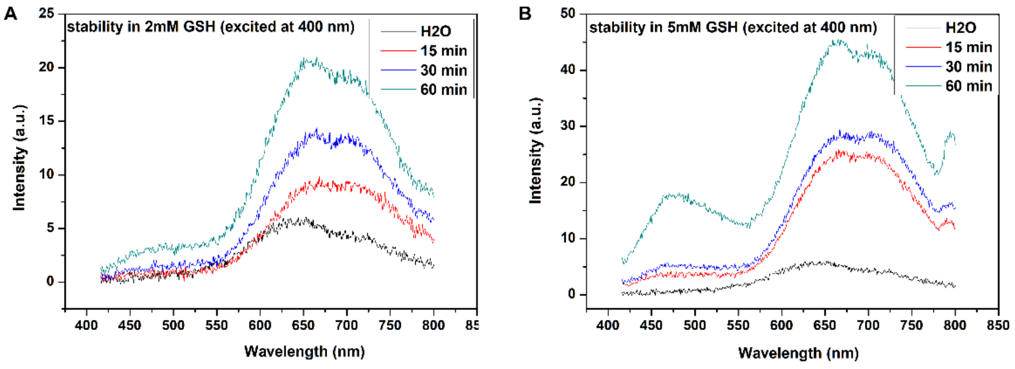

Figure S5. Time-stability of AgNCs in GSH. Emission spectrum of AgNCs excited at 400 nm in the presence of 2 mM GSH **(A)** and 5 mM GSH **(B)**.

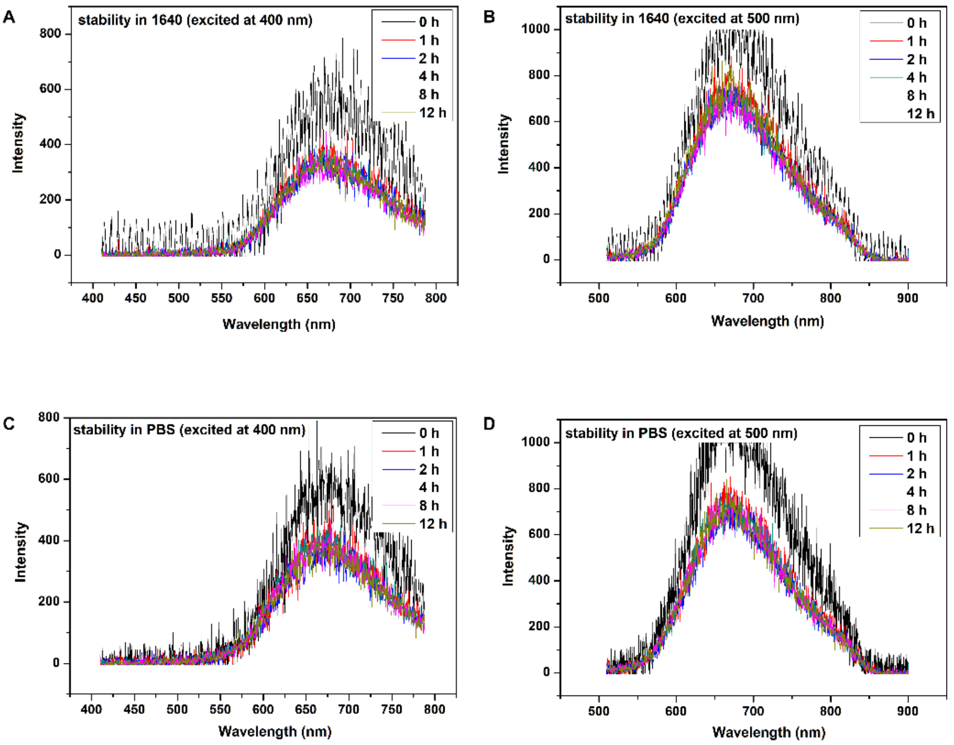

Figure S6. Biological stability of AgNCs in RPMI-1640 medium and PBS. Emission spectrum of AgNCs excited at 400 nm in RPMI-1640 medium **(A)** and PBS **(C)**. Emission spectrum of AgNCs excited at 500 nm in RPMI-1640 medium **(B)** and PBS **(D)**.

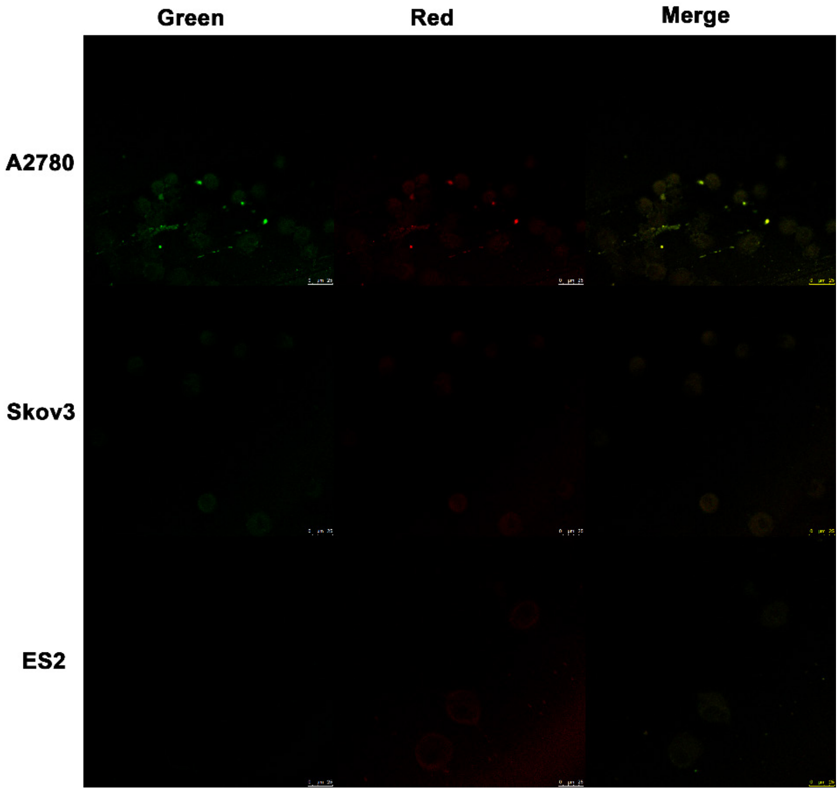

Figure S7. Confocal images of cellular ROS with AgNCs in ovarian cancer cell lines (A280, Skov3, and ES2).

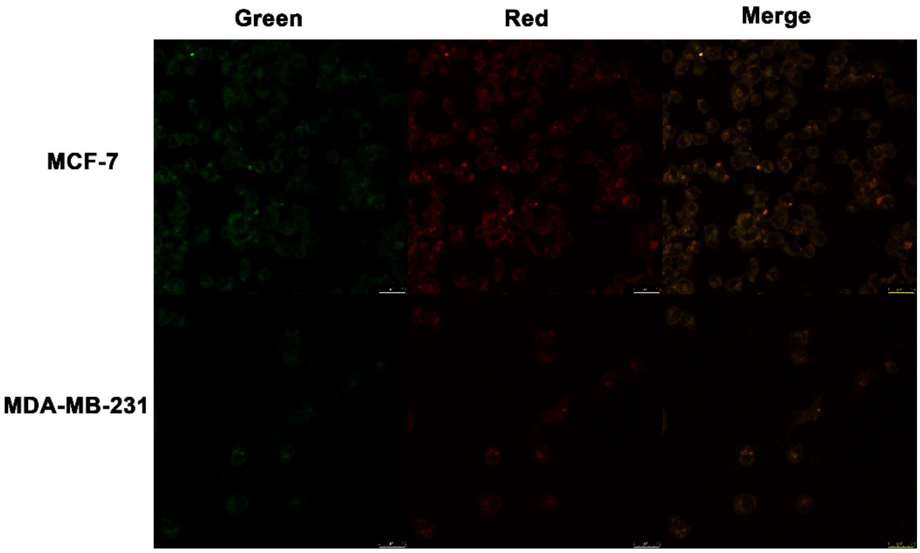

Figure S8 Confocal images of cellular ROS with AgNCs in breast cancer cell lines (MCF-7 and MAC-MB-231).

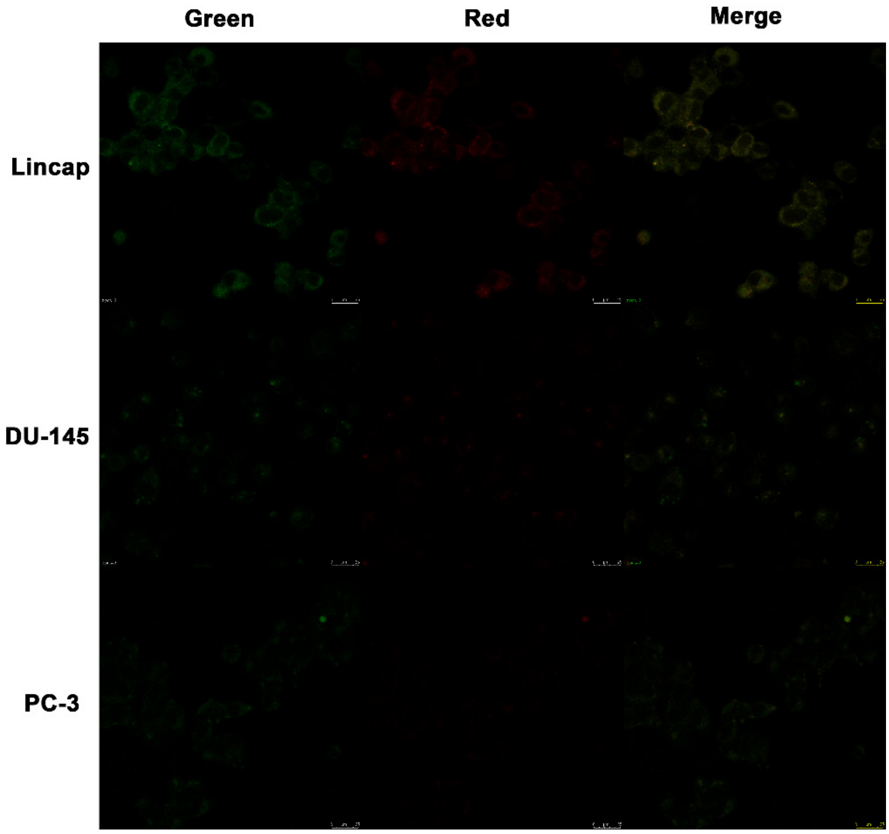

Figure S9. Confocal images of cellular ROS with AgNCs in prostate cancer cell lines (Lincap, DU-145, and PC-3).

Table S1. The relationship among tumor histologic types, genetic alterations and ROS species

Cell line	Tumor histologic type	Genetic alterations	Fluorescence of AgNCs	ROS detection regents	Migration activity	Malignant phenotype transformation
FTC133	FTC	PTEN (R130X +/+)	Green emission↑<br>Red emission↓	H_2O_2	Weak	-
BCPAP	PTC	BRAF V600E+/+	Green emission↑<br>Red emission↓↓	H_2O_2,<br>slight O_2^-, and •OH	Moderate	MMP-9 activity
OCUT-2	ATC	BRAF V600E+/-<br>PIK3CA	Faint fluorescence	H_2O_2,<br>enhanced O_2^-,<br>•OH	Strong	MMP-9 activity<br>EMT activity
TPC1	ATC	RET/PTC1	Faint fluorescence	H_2O_2,<br>abundant O_2^-,	Strong	MMP-9 activity<br>EMT activity

	•OH

*FTC, follicular differentiated thyroid cance; PTC , papillary thyroid carcinoma; ATC, anaplastic thyroid carcinoma; +/-, heterozygous mutation; +/+, homozygous mutation; X: stop codon
